# Interleukin-33 Amplifies Human Mast Cell Activities Induced by Complement Anaphylatoxins

**DOI:** 10.3389/fimmu.2020.615236

**Published:** 2021-02-01

**Authors:** Peter W. West, Rajia Bahri, Karen M. Garcia-Rodriguez, Georgia Sweetland, Georgia Wileman, Rajesh Shah, Angeles Montero, Laura Rapley, Silvia Bulfone-Paus

**Affiliations:** ^1^ Lydia Becker Institute of Immunology and Inflammation, School of Biological Sciences, Faculty of Biology, Medicine and Health, University of Manchester, Manchester Academic Health Science Centre, Manchester, United Kingdom; ^2^ Manchester University NHS Foundation Trust, Wythenshawe Hospital, Manchester, United Kingdom; ^3^ Adaptive Immunity, GlaxoSmithKline, Stevenage, United Kingdom

**Keywords:** mast cells, complement C3a, complement C5a, complement, degranulation, interleukin-33, cytokine

## Abstract

Both, aberrant mast cell responses and complement activation contribute to allergic diseases. Since mast cells are highly responsive to C3a and C5a, while Interleukin-33 (IL-33) is a potent mast cell activator, we hypothesized that IL-33 critically regulates mast cell responses to complement anaphylatoxins. We sought to understand whether C3a and C5a differentially activate primary human mast cells, and probe whether IL-33 regulates C3a/C5a-induced mast cell activities. Primary human mast cells were generated from peripheral blood precursors or isolated from healthy human lung tissue, and mast cell complement receptor expression, degranulation, mediator release, phosphorylation patterns, and calcium flux were assessed. Human mast cells of distinct origin express constitutively higher levels of C3aR1 than C5aR1, and both receptors are downregulated by anaphylatoxins. While C3a is a potent mast cell degranulation inducer, C5a is a weaker secretagogue with more delayed effects. Importantly, IL-33 potently enhances the human mast cell reactivity to C3a and C5a (degranulation, cytokine and chemokine release), independent of changes in C3a or C5a receptor expression or the level of Ca^2+^ influx. Instead, this reflects differential dynamics of intracellular signaling such as ERK1/2 phosphorylation. Since primary human mast cells respond differentially to anaphylatoxin stimulation, and that IL-33 is a key regulator of mast cell responses to complement anaphylatoxins, this is likely to aggravate Th2 immune responses. This newly identified cross-regulation may be important for controlling exacerbated complement- and mast cell-dependent Th2 responses and thus provides an additional rationale for targeting anti-IL33 therapeutically in allergic diseases.

## Introduction

Both mouse and human MCs express complement anaphylatoxin, C3a and C5a, receptors (1–4). Both C3a and C5a bind to G protein-coupled receptors that belong to the superfamily of rhodopsin-like receptors (5, 6). However, the modalities of C3a and C5a receptor-binding and activation differ. While the C3a C-terminal peptide contains virtually all of the biological function, mediating C3a binding and signaling through its receptor C3aR1, C5a C-terminal peptides display only some biological activity, and in addition discontinuous regions of C5a contribute to C5aR1 and C5aR2 interaction with the last 6 or 7 C-terminal residues being functionally selective for C5aR2 (7, 8).

The level of receptor expression seems to be both species and tissue specific (9). Skin MCs express C3aR1 (10) and C5aR1 while lung, uterine and tonsil MCs have been described neither to express C5aR1 nor C5aR2 (11). C3aR1 expression was also found in airway smooth muscle MCs (12). C3aR1 and C5aR1 have been detected in the human mast cell lines HMC-1 and LAD2 (1, 13–19). C3a and C5a have been shown to induce intracellular calcium Ca^2+^ mobilization, causing degranulation, and cytokine and chemokine secretion (16, 20). Furthermore, the use of C3ar1^-/-^ and C5ar1^-/-^ MCs in passive cutaneous anaphylaxis (PCA) mouse models and C5ar1^-/-^ mice in oral food allergy models has demonstrated the contribution of anaphylatoxin signaling pathways in IgE-induced MC activation (21, 22).

IL-33 is released by fibroblasts (23), endothelial cells (24), and epithelial cells upon environmental injury (25–27). It is a potent MC activator that plays a pro-inflammatory role in several diseases such as asthma (28) atopic dermatitis (29–31), psoriasis, allergies (32, 33) and inflammatory arthritis (34). IL-33 has a predominantly amplifying immunomodulatory role in diverse immune cell subsets (35) and has been shown to potentiate IgE-mediated MC responses (36) by inducing cytokine production, and increasing degranulation. However, prolonged treatment with IL-33 has also been found to suppress MC activities (37, 38). Thus, this underlines the multifaceted properties of this cytokine.

Complement anaphylatoxins are expressed in lung and skin epithelia (39–41) alongside IL-33 and are co-produced in acute and chronic inflammatory reactions (28, 42–46), where MCs are likely to contribute to disease. Therefore, in this study sought to mimic the early inflammatory response by investigating whether C3a and C5a differentially activate primary human MCs (hMCs), and whether IL-33 regulates C3a/C5a-induced MC activities. We provide evidence that C3a is a potent degranulation inducer while C5a is a weaker agonist with more delayed effects. Furthermore, IL-33 potently increases the MC reactivity to C3a and C5a by alterations in intracellular signaling.

## Methods

### Human Tissue and Blood Samples

Human biological samples were sourced ethically and their use was in accord with the terms of informed consent under IRB approved protocols in accord with the declaration of Helsinki. Anonymized samples of uninvolved lung tissue from three donors were obtained from the Manchester Allergy Respiratory and Thoracic Surgery (ManARTS) biobank (NHS REC Ref 15/NW/0409) from patients undergoing surgical resection for suspected or confirmed lung cancer. Peripheral blood NC24 leukocyte cones obtained from anonymous healthy donors were obtained from NHS blood and transplant (Manchester, UK) and used in accordance with a protocol approved by the University of Manchester research ethics committee (UREC ref 2018-2696-5711).

### Human Mast Cell Isolation and Culture

Peripheral blood derived mast cells were generated as previously described (47, 48) and detailed in the online supplement. Human lung mast cells (hLMC) were obtained according to a modified WEMP protocol described in detail elsewhere (49) and in the online supplement. A total of 65 individual donors were used in this manuscript.

### Statistical Analysis

Data are presented as mean ± SEM or SD of independent experiments, as indicated in the figure legends. Data were analyzed using Wilcoxon matched-pairs signed rank test, paired t-test, one-way or two-way ANOVA with indicated post-hoc comparison test. All data were analyzed using Graphpad Prism software v7.00 or v8.00 with significant differences indicated by * = p<0.05, ** = p<0.01 *** = p<0.0 1, **** = p<0.0001. Additionally flow-cytometry histograms comparing FMO/Isotype to specific antibody labelled samples from individual donors were analyzed using population comparison tool in FlowJo v10.7.1. Where T(X)>4 indicates probability that populations are significantly different with >99% probability. Detailed summary statistics are presented in **Supplementary Table 4**.

### Other Methods

Other methods are described in the online supplement.

## Results

### Complement Receptor Expression and Ligand-Induced Modulation of Human Mast Cells

**Figure 1:**
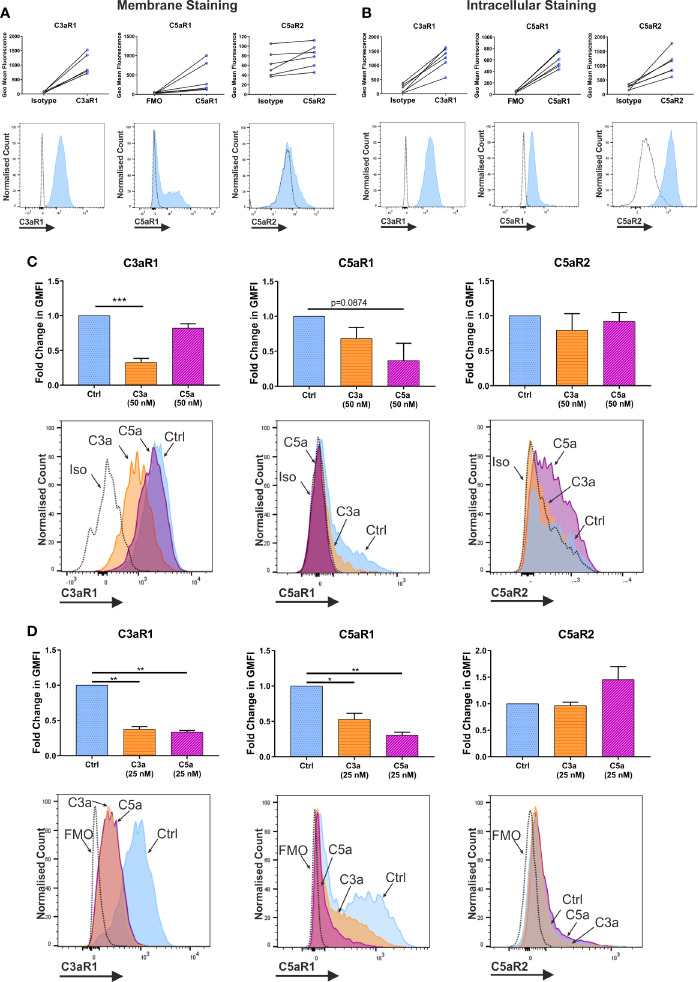
Expression and modulation of complement receptors. Expression of anaphylatoxin receptors shown by geometric mean fluorescence, **(A)** on the membrane; and **(B)** in permeabilized cells, with representative histograms. Antibody stained samples are shown in blue with control samples in grey. Data are n=6 of six independent experiments in six donors. **(C)** Change in membrane derived geometric mean fluorescence (GMFI) indicative of expression after 1 h incubation with ligand or media control treatment. Data are mean ± SEM of n=6 independent experiments from six donors, with representative histograms. **(D)** Change in membrane derived geometric mean fluorescence (GMFI) indicative of expression after 24 h with ligand or media control, and 8 h in media alone. Data are mean ± SEM of n=3 independent experiments from nine pooled donors. Significant differences are indicated by * = p<0.05, ** = p<0.01, *** = p<0.001 (One-way ANOVA with Dunnett’s post-test). Summary statistics are presented in the supplementary material.

Activated murine MCs and human MC lines have been described to react to complement anaphylatoxins and express complement receptors (1). Indeed, human skin exhibits a wheal and flare reaction to C5a (50). To investigate hMC complement receptors, we have utilized a well-defined model of *in vitro* blood derived hMCs (47, 48) (**Supplementary Figure 1A**). Resting hMCs expressed high levels of membrane C3aR1, variable, but intermediate levels C5aR1 and low heterogeneous levels of C5aR2, showing a wide expression peak (**Figure 1A**). However, the expression was comparable between all three receptors when using intracellular analysis. (**Figure 1B**). While the expression of inhibitory complement receptors CD46, CD55, and CD59 was found using both membrane and intracellular staining techniques (**Supplementary Figure 1B**), the complement receptors CD11b, CD35, and CD93 were absent (**Supplementary Figure 1C**).

To further address whether C3a and C5a affect complement receptor expression and whether cross-reactivity exists between the ligands, hMCs were incubated with either C3a or C5a and expression evaluated. While 1 h incubation with C3a consistently downregulated C3aR1 on the membrane, C5a binding showed only a non-significant trend towards down-modulating C5aR1 (**Figure 1C**). Although the donors used in this experiment appeared to have slightly lower natural expression of C5aR1. Furthermore, while the total expression (in permeabilized cells) of C5aR1 remained unchanged on anaphylatoxin exposure, C3aR1 was decreased by C3a stimulation (**Supplementary Figure 2A**). The expression of C5aR2 was constitutive and not altered in any condition investigated (**Figure 1**, and **Supplementary Figure 2A**) although C3a induced a small downregulation inconsistently. However, a 24 h long exposure of hMCs to C3a and C5a, followed by resting the cells, showed that both anaphylatoxins cross-modulate C5aR1 and C3aR1, respectively (**Figure 1D**). Mast cell degranulation induced upon FcϵRI engagement by IgE/α-IgE stimulation was used as a control and found to reduce the expression of both receptors C3aR1 and C5aR1 (**Supplementary Figure 2C**).

**Figure 2:**
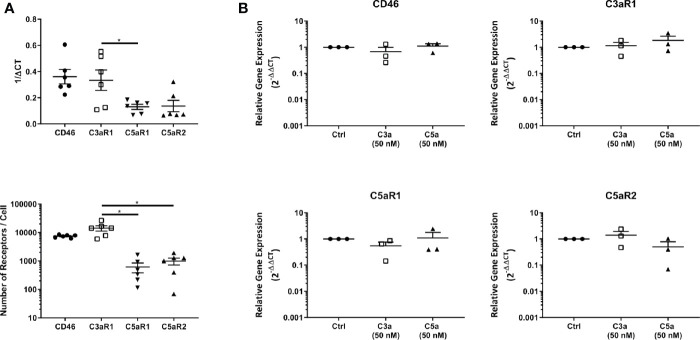
Baseline expression, quantitation and modulation of complement receptors. **(A)** Complement receptor expression in untreated mast cells determined by qPCR (above) and fluorescence quantitation (below). Data are mean ± SEM of n=6 experiments from 9 pooled donors and 6 individual donors respectively. **(B)** qPCR expression of complement receptors 8 h after addition of C3a (50 nM), C5a (50 nM), or media control. Data are mean ± SEM of n=3 independent experiments from three donors. Significant differences are indicated by * = p<0.05 (ANOVA with Dunnett’s post-test). Summary statistics are presented in the **Supplementary Material**.

C3aR1, and C5aR1 or C5aR2 expression at the cell membrane were confirmed with fluorescence quantitation and found to reproduce the transcript level data (**Figure 2A**). Furthermore, the expression of C3aR1 was significantly higher than that of C5aR1 at the transcript level and significantly higher than C5aR1 and C5aR2 at the protein level. Furthermore, C3aR1, C5aR1, and C5aR2 transcriptional expression was not altered on C3a and C5a stimulation (**Figure 2B**).

In summary, C3aR1, C5aR1, and C5aR2 are constitutively but differentially expressed on resting hMCs. C3a ligand binding, possibly by internalization, rapidly and significantly downregulated C3aR1 after 1 h while C3a reduced C5aR1 fluorescence at the cell membrane, but not significantly so. However, at this early time point, C5a specifically, but not significantly, appeared to affect its cognate receptor C5aR1, while inducing negative feedback on C3aR1 at a later time point. Thus, these data suggest C5aR2 does not participate in MC activation, but both C3aR1 and C5aR1 engagement carry cross-modulatory activities.

### C3a Induces a Potent MC Degranulation

**Figure 3:**
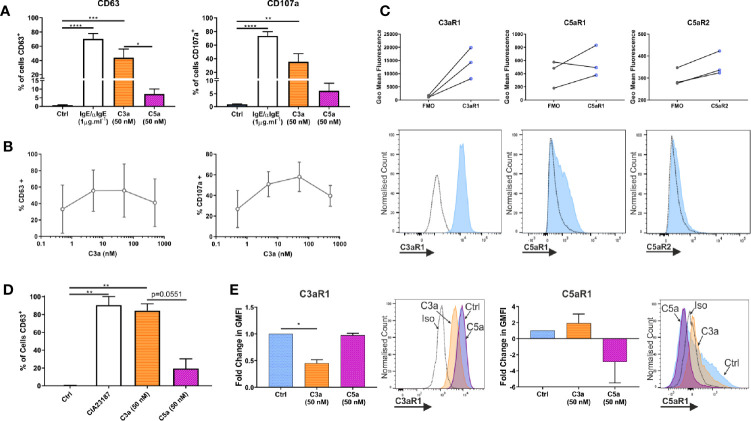
Complement anaphylatoxins differentially induce degranulation. **(A)** Degranulation measured by externalization of CD63 or CD107a after treatment for 1 h with ligands as shown. Data are mean ± SEM of n=7 independent experiments from 7 separate donors. **(B)** Degranulation, by CD63 (n=3) or CD107a (n=2) in response to C3a (0.5–500 nM). Data are mean ± SD. **(C)** Receptor expression based on geometric mean fluorescence, with specific antibodies (blue) vs FMO controls (grey) on hLMCs. Data are n=3 of independent experiments from 3 donors with representative histograms. **(D)** Degranulation of hLMCs measured by percentage of cells in positive gate for CD63 after 1 h treatment with ligands as indicated. Data are mean ± SEM, of n=3 experiments from three donors. **(E)** Changes in complement receptor C3aR1 or C5aR1 expression due to anaphylatoxin treatement for 1 h with C3a (50 nM) (orange bar with horizontal stripe) or C5a (50 nM) (magenta bas with diagonal stripe). Data are mean ± SEM of three experiments from three donors with representative histograms showing membrane expression in response to ligands (as labelled). Differences indicated * = p<0.05, ** = p<0.01, *** = p<0.001, **** = p<0.0001 (One-way ANOVA with Dunnett’s or Tukey’s post-tests). Summary statistics are presented in the supplementary material.

Both anaphylatoxins, C3a and C5a, are described to modulate MC activities although evidence comes largely from cell lines HMC-1 and LAD2 (1, 18, 51). Anaphylatoxins are generated by complement activation, with C5 being downstream of C3 in the cascade. However, their specific complementary or interdependent role on primary hMCs is still unclear. To determine the functionality of the anaphylatoxin receptors, hMCs were stimulated with C3a and C5a (50 nM), and degranulation assessed by externalization of CD63 and CD107a. IgE-induced degranulation was used as a positive control (**Figure 3A**, and **Supplementary Figure 3A**). Upon C3a exposure (0.5–500 nM) the % of degranulating hMCs (CD63^+^ or CD107a^+^) increased to a max of 55.8 ± 32.3 and 58.0 ± 14.24 respectively reaching a plateau at between 5–50 nM (**Figure 3B**). The degranulation response achieved to C5a was 23.6 ± 18.5% and 37.8 ± 26.2% at 50 nM concentration. Even at 500 nM this was not exceeded, with responses of 22.3 ± 20.2% and 37.3 ± 30.4% CD63^+^ and CD107a^+^ cells respectively (**Supplementary Figure 3B**).

To define whether the findings above are comparable between peripheral blood-derived and tissue MCs, the latter (CD45^+^, CD117^+^, FcϵRI^+^ and lineage negative; **Supplementary Figure 3C**) were isolated from lung tissue. Human lung MCs (hLMCs) expressed high levels of C3aR1 and, in two out of three donors, lower levels of C5aR1 and R2 (**Figure 3C**). Furthermore, hLMCs showed a high level of degranulation in response to C3a (84.3 ± 7.6% CD63^+^ cells) almost comparable to potent calcium ionophore stimulus (CIA23187), used as positive control (90.5 ± 9.7% CD63^+^ cells), while the effect of C5a stimulation remained negligible (19.5 ± 10.7% CD63^+^ cells) (**Figure 3D** and **Supplementary Figure 3D**). Moreover, as in blood derived hMCs, the exposure of hLMCs to C3a significantly downregulated C3aR1 and non-significantly affected C5aR1, while the C3aR1 expression was not affected by C5a stimulation (**Figure 3E**).

These data support the concept that C3a and C5a exert distinct functional activities on hMCs with C3a predominantly inducing degranulation. Since these findings were validated in isolated hLMCs they are likely to be relevant to complement anaphylatoxin receptors *in vivo*.

### Mast Cell Sensitivity and Reactivity to C3a and C5a Are Distinct

The importance of Ca^2+^ influx in mast cell activation and degranulation is widely accepted and is triggered by the depletion of Ca^2+^ stores in the endoplasmic reticulum, which activates Ca^2+^ channels at the plasma membrane (52).

**Figure 4:**
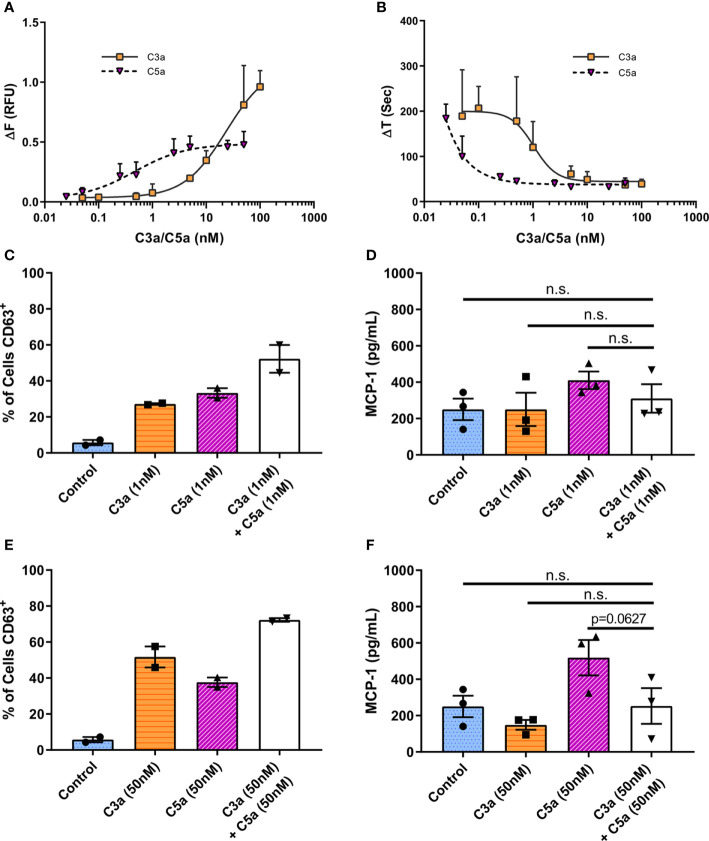
Differential and additive effects of complement anaphylatoxins. **(A)** Change in Fura-2AM specific ratiometric fluorescence induced by C3a (0.05–100 nM) and C5a (0.025–50 nM). EC_50_ [C3a] = 23.57 nM, EC_50_ [C5a] = 0.4723 nM. **(B)** Time in seconds taken to reach maximum fluorescence (ΔT). Data are mean **±** SD of three independent experiments on three donors. **(C–F)** Degranulation measured by CD63 externalization at 1 h after treatment with ligands as indicated, presented as percentage of cells in the CD63 positive gate **(C, E)**, and MCP-1 production at 8 h **(D, F)** after addition of C3a (1 or 50 nM) C5a (1 or 50 nM) of both C3a + C5a (1 or 50 nM) for 8 h. Data are mean ± SEM of n=2 **(C, E)** or 3 **(D, F)** from a total of six or nine pooled donors respectively. Summary statistics are presented in the **Supplementary Material**.

To assess C3a and C5a induced Ca^2+^ mobilization, we utilized the calcium-sensitive ratiometric fluorescent dye Fura-2. When hMCs were stimulated with C3a or C5a (**Figure 4**), intracellular calcium increased at significantly lower concentrations of C5a (EC_50_ = 0.4723 nM) compared to C3a (EC_50_ = 23.57 nM) (**Figure 4A**). The time taken to reach peak fluorescence was consistent with concentration dependent alteration in fluorescence maxima. Both anaphylatoxins exhibited similar, fast Ca^2+^ release, reaching peak fluorescence within 30 s at optimum ligand concentrations, taking longer to reach peak at lower ligand concentrations (**Figure 4B**). This anaphylatoxin-induced Ca^2+^ influx was dependent on extracellular calcium (**Supplementary Figure 4**). While C5a stimulation induced a modest intracellular calcium release with plateau at relative low concentrations (>2.5 nM), high C3a concentrations (between 10 and 100 nM) caused a larger and substantial rise in Ca^2+^.

These data suggest that low concentrations of C5a can initiate a rapid Ca^2+^ influx while only higher C3a concentrations stimulate the influx of intracellular Ca^2+^ to levels that cause productive hMC degranulation.

### C3a and C5a Display Limited Synergism in Human Mast Cells

To assess C3a and C5a synergism in the modulation of MC activities, C3a and C5a were added simultaneously to cells at low (**Figures 4C, D**) or maximal (**Figures 4E, F**) concentrations. The co-application of both anaphylatoxins to hMCs showed a very modest additive effect on overall degranulation as measured by % of CD63^+^ hMCs (**Figures 4C, E**), with an antagonistic effect on maximal Ca^2+^ release, although a small (<25%) synergistic effect at lower concentrations and effect sizes (**Supplementary Figure 5**). The release of MCP-1 induced by C5a hMC stimulation was not significantly increased or decreased by the addition of C3a (**Figures 4D, F**, p=0.1890 & p=0.0627). Incubation of hMCs with C3a and C5a (50nM) combined, downregulated both C3aR1 and C5aR1 surface expression, and had no effect on C5aR2 (**Supplementary Figure 6B, D**, **F**). Interestingly, simultaneous application of C3a and C5a, or C5a alone, at low (1nM) concentrations effectively reduced C5aR1 membrane expression (**Supplementary Figure 6C**) but had no significant effect on C3aR1 or C5aR2 (**Supplementary Figure 6A**, **E**). These data suggest that C5a may be additive to C3a induced activities at sub-maximal C3a concentrations but that C3a may not be additive to C5a driven responses such as MCP-1 production. However, the findings indicate that the expression of C5aR1 is fine-tuned at low anaphylatoxin concentrations.

### IL-33 Potentiate Human Mast Cell Responses to Complement Anaphylatoxins

**Figure 5:**
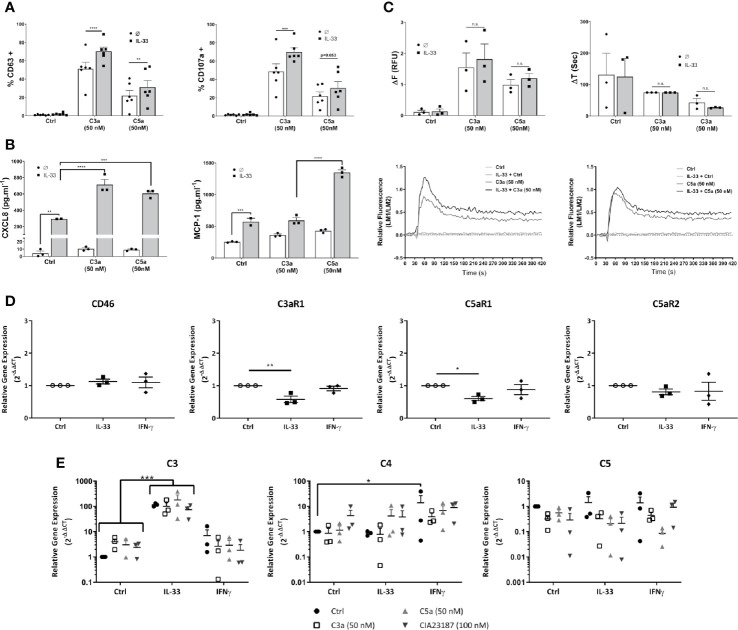
IL-33 induced potentiation of responses to complement anaphylatoxins. Cells treated for 24 h with control, IL-33 (50 ng.ml^-1^), or IFNγ (50 ng.ml^-1^), followed by activation as indicated with media control C3a (50 nM) or C5a (50 nM) or CIA23187 (100 nM) **(A–C, E)** showing; **(A)** degranulation 1 h post activation, measured by externalization of CD63 or CD107a, given as percentage of cells in the positive gate for CD63 or CD107a (% +); **(B)** CXCL8 and MCP-1 production and **(E)** complement gene expression 8 h post treatment with anaphylatoxins or CIA23187; and **(C)** calcium flux showing change in FURA-2AM specific fluorescence (ΔF) or time taken to reach maximum fluorescence (ΔT) induced by C3a (50 nM) or C5a (50 nM) with representative examples. **(D)** Gene expression after 24 h of treatment with IL-33 (50 ng.ml^-1^) or IFN (50 ng.ml^-1^). Data are **(A)** mean ± SEM of n=6 experiments from a total of eight donors, **(B)** mean ± SEM of n=3 experiments from three donors (representative of n=6 experiments from six donors), **(C)** mean ± SEM of three experiments from three donors, **(D)** mean ± SEM of n=3 independent experiments from a total of 11 donors and **(E)** mean ± SEM of three independent experiments from three donors. * = p<0.05, ** = p<0.01 *** = p<0.0 1, **** = p<0.0001 as determined by **(A, C)** 2-way ANOVA with Sidak’s post-test, **(B)** 2-way ANOVA with Tukey’s post-test, **(D)** 1-way ANOVA with Sidak’s post-test or **(E)** 2-way ANOVA with Tukey’s post-test. Summary statistics are presented in the **Supplementary Material**.

Cytokine conditioning can mimic the inflammatory tissue environment (53–56). To explore whether epithelial-derived, pro-inflammatory cytokines regulate the activities of anaphylatoxins on hMCs in tissue, we exposed hMCs for 24 h to IL-33, IFN-γ or IL-4 prior to stimulation with C3a or C5a. IL-33 significantly increased C3a- and to a lower extent C5a-induced degranulation as measured by % of CD63^+^ or CD107a^+^ cells (**Figure 5A**). Furthermore, IL-33 significantly increased secretion of CXCL8 by hMCs stimulated with both C3a or C5a and MCP-1 when exposed to C5a only (**Figure 5B**). In contrast to IL-33, IL-4 and IFN-γ conditioning increased MCP-1 secretion by hMCs on stimulation with both C3a and C5a but had no effect on CXCL8 release (**Supplementary Figures 7A–D**). In keeping with other published data (57), we also observed IL-13 production in IL-33 conditioned cells (data not shown). Although IL-33 conditioning augmented the Ca^2+^ flux response to C3a and C5a (**Figure 5C**), the increase is modest and non-significant. Thus, these results suggest that the consistent and significant increase in complement induced hMC degranulation and mediator release caused by IL-33 conditioning cannot be explained solely by an increase in Ca^2+^ release.

Next, we investigated whether degranulation or mediator secretion was augmented by production of intrinsic complement, as described for mast cell line HMC-1 (56), or an upregulation of anaphylatoxin receptors induced by cytokine preconditioning. Complement mediators, C3, C4 and C5 as well as C3aR1, C5aR1 and C5aR2 transcript analysis was performed in hMCs treated for 24 h with either IL-33 or IFN-γ and stimulated 8 h with either C3a or C5a. Neither IL-33 nor IFN-γ substantially increased complement receptor gene expression and in fact C3aR1 and C5aR1 were significantly reduced by IL-33 (**Figure 5D**). Furthermore, while IL-33 upregulated C3, IFN-γ increased C4 transcript. However, no increase in anaphylatoxin release could be detected (**Figure 5E**, and **Supplementary Figure 7E**) and IL-33 conditioning did not alter complement receptor protein levels on the cell surface (**Supplementary Figure 7F**).

Altogether, these data demonstrate that IL-33 is a key modulator of complement-mediated activities on hMCs, potentiating specific features of C3a and C5a. These effects are independent from IL-33-regulation of complement receptors.

### IL-33 Alters the Intracellular Signaling Dynamic of Human Mast Cells to C3a and C5a

To better understand the mechanism guiding the IL-33-dependent effect of anaphylatoxin-mediated activities on hMCs, we compared the kinetics of C3a and C5a-induced tyrosine phosphorylation of ERK1/2 between IL-33 conditioned and untreated hMCs, observing ERK1/2 activation as an indicator of signaling of the C3aR1 and C5aR1 pathway.

**Figure 6:**
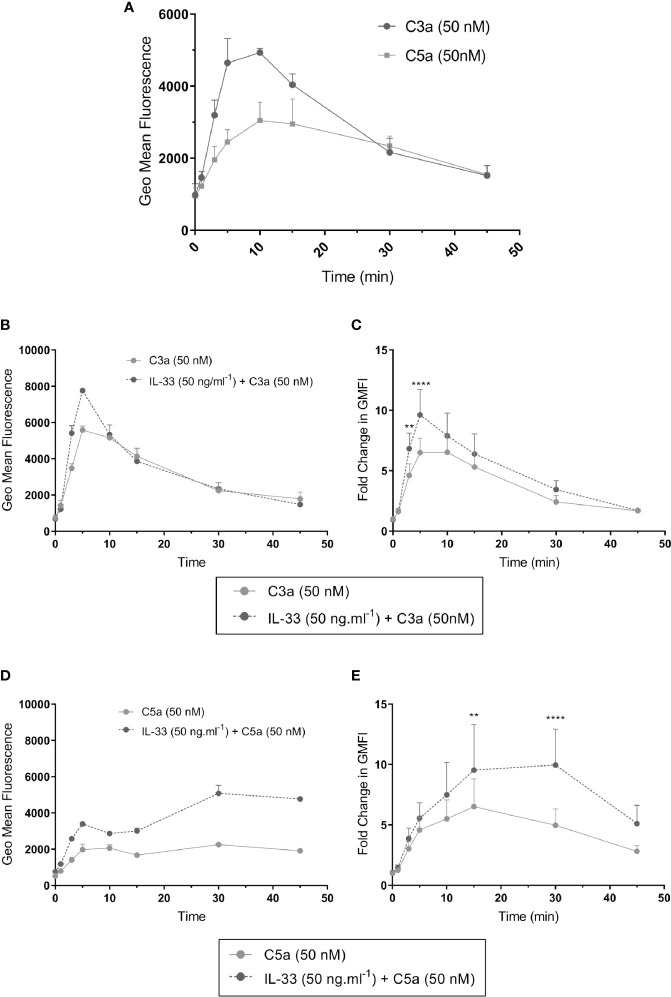
ERK1/2 activation by complement anaphylatoxins. ERK1/2 phosphorylation measured by change in phosphor-ERK specific fluorescence (geometric mean fluorescence intensity, GMFI) after treatment for times shown (0, 1, 3, 5, 10, 15, 30, 45 min) with **(A)** C3a (50 nM) or C5a (50 nM) alone or **(B–E)** after additional pre-conditioning with IL-33 (50 ng.ml^-1^) for 24 h and then activation with C3a (50 nM) or C5a (50 nM). Data are mean ± SEM of three experiments **(A)**, replicates of a representative example **(B, D)**, or seven independent experiments from seven donors **(C, E)**. ** = p<0.01, **** = p<0.0001 (ANOVA with Sidak’s post-test). Summary statistics are presented in the **Supplementary Material**.

The profile of ERK1/2 activation induced by C3a and C5a differed. C3a induced approximately 5 fold increase in ERK1/2 specific fluorescence, indicating strong activation of the phosphorylation cascade, with a peak around 5 min. C5a showed weaker and retarded ERK1/2 phosphorylation (**Figure 6A** and **Supplementary Figure 8**). Compared with controls, IL-33-conditioned hMCs showed 1.43 ± 0.36 (mean ± SD, n=7) fold greater phosphorylation of ERK1/2 at 5 min of C3a exposure (**Figures 6B–E**). However, while the C3a-induced peak of ERK1/2 phosphorylation diminished rapidly to baseline levels irrespective of the cell exposure to IL-33, C5a-induced increase in ERK1/2 phosphorylation was maintained in an IL-33 dependent manner (**Figures 6D, E**), reaching 1.95 ± 0.66 (mean ± SD, n=7) fold greater phosphorylation at 30 min.

These data suggest that C3aR1 and C5aR1 signaling differ in intensity and dynamics, which influence the cell response toward degranulation or mediator secretion. The epithelial-derived cytokine IL-33 potently amplifies the hMC response to anaphylatoxins and introduces positive feedback on C3 expression, thus maintaining the specificities in C3a and C5a-induced activities.

## Discussion

We provide evidence that the complement anaphylatoxins C3a and C5a induce distinct activities in hMCs of different origin. While C3a is a potent and rapid degranulation inducer, C5a is a weaker agonist with more delayed effects. Furthermore, IL-33 potentiated these polarised responses, increasing the number of cells degranulating in response to C3a and MCP-1 secretion in response to C5a. The potentiating effect of IL-33 seems not dependent on the upregulation of the complement receptors but in altering the intensity or duration of downstream signaling.

C3a induced degranulation has been described in MCs obtained from different organisms and organs, in cell lines and primary cells (1, 16, 21, 51). Yet the reports on the type of both C3a- and C5a-induced MC activities are various and controversial (58, 59). This may be attributable to the level of expression of the receptors in human MC subtypes (11, 60). In our study, we demonstrate that effects observed in MCs differentiated from human blood progenitors, reproduce the outcomes obtained in the *ex-vivo* stimulation of primary human lung MCs. Furthermore, we found that the level and specificities of receptors expression was comparable between the two hMCs populations. Our data support many findings from the LAD2 cell line (16), particularly in relation to the relative potency of C3a and C5a. Oskeritzian et al. (60) described C5aR1 expression on a subset of CD117^+^ cells, in human lung. Our findings concur, inasmuch as we detected a similar percentage of C5a responsive MCs in the overall population (11% vs 7.22%). Therefore, in this context, the results originated using blood-differentiated MCs, which were valuable for researching the mechanistic aspects of our study were validated in tissue MCs.

Both anaphylatoxins have been described to bind their respective cognate receptors with high affinity (61). MC activation mediated by C3a differed from C5a in the amplitude of the induced degranulation, with a potent and consistent effect of C3a and a mild response to C5a. The combined stimulation by the two anaphylatoxins, even at low concentrations shows only limited synergism. Instead, these results indicate that the C3a and C5a exert a reciprocal containing effect. Indeed, we have shown that not only does the process of degranulation decrease the expression of the C3aR1 on the membrane, probably due to receptor internalization, but also that MC stimulation by C3a can downregulate C5aR1. This would suggest that C3a has functionally limiting effects over C5a responses.

Our data show C5a can initiate a rapid Ca^2+^ influx at low concentrations. In contrast, only higher C3a concentrations mobilize intracellular Ca^2+^ to levels that produce hMC degranulation. Our findings reproduce the absence of C5a dose-dependency at high concentrations that has been reported in mast cells lines and basophils (16, 62). Gaudenzio et al. demonstrated rapid Ca^2+^ peak induced by both C3a and C5a, resulting in rapid secretion of individual secretory granules (63). The rapid calcium release; extent of C3a induced Ca^2+^ flux being larger than C5a; and the time-profile of the calcium flux, were similar in our data. We also observed, the prolonged C5a induced calcium release at 2–3 min seen by these authors. These data are consistent with a lower, slower but prolonged effect of C5a on hMC activity. Slight differences in the extent of degranulation between our data may be accounted for by concentration, source of complement as well as natural donor variability. As can be seen in our data, supramaximal C3a concentrations tended to inhibit degranulation by around 20% and receptor internalization may limit C3a responsiveness (19) and degranulation. We used human serum-derived complement, whereas Gaudenzio et al. used recombinant forms. Differential receptor engagement might then alter the downstream activation. In addition, we performed experiments in a large number of donors and noted variability particularly in relation to C5a, which is evident in our data. Therefore, the observations by Gaudenzio et al. in response to C5a are within the margin of this variability.

Interestingly, our findings demonstrate that IL-33 is a key modulator of complement-mediated activities of hMCs, thereby potentiating C3a and C5a specific features such as degranulation and mediator secretion. Furthermore, on IL-33 pretreatment, C5a, not C3a, produced the most MCP-1. This is different from the effect of IL-33 on CXCL8 secretion that is equally increased. Therefore, our studies show that MC activation induced by C3a and C5a support distinct MC functions: a broad pro-inflammatory program versus chemoattractant activities, respectively. Our findings from primary cells clarify the existing literature in this regard, since although in the HMC-1 cell line, C3a was reported to be the primary inducer of MC MCP-1 production (14), in LAD2 cells both C3a and C5a have been shown to induce MCP-1 (1). However, the recombinant form of C5a used in previous studies may have differential activating potential on the receptor (64).

To investigate the dynamic of complement anaphylatoxin-induced MC activation after IL-33 pretreatment, we observed ERK1/2 phosphorylation upon C3a and C5a stimulation. The peak in ERK1/2 phosphorylation, appearing within the first 30 min of stimulation, has been reported to indicate anaphylatoxin receptor activation and is a key early response pathway in MC signaling linked to IL-8 and MCP-1 production (1, 14, 52, 65). C3a stimulation produces a higher early peak of ERK1/2 phosphorylation compared to C5a, but also declines rapidly. Our studies indicate IL-33 does not regulate membrane expression of anaphylatoxin receptors in the acute phase, but instead potentiates the intracellular signaling. Although down-regulation of gene expression supports longer term reduction in MC responsiveness (37). The effects we observed might be mediated through GRK- induced receptor phosphorylation and β-arrestin recruitment, shown to impact complement induced degranulation, ERK1/2 phosphorylation and MCP-1 production (19, 66) since IL-33 has been shown to modulate GRK2 in other cell types (67). Additionally, more sustained ERK1/2 activation by fMLP in HMC-1 cells in known to influence MIP-1β and MCP-1 production (68). However, in the case of C5a, β-arrestin recruitment is not thought to impact on ERK1/2 signaling (64). Indeed, there are multiple levels at which MC degranulation, calcium flux and ERK1/2 might be modulated in MCs (69). IL-33 amplifies the observed differences and translates them in distinct functional properties such as the increase in MCP-1 secretion induced by C5a. Whether receptor crosstalk allows the augmented signaling at the cell membrane, through increased availability of intracellular signaling adaptor proteins, or the synergistic action of distinct signaling pathways, along with how these factors might separately influence MC degranulation or cytokine secretion, remains to be determined.

Clinically, the complement system and IL-33 have been increasingly linked to allergic conditions (59, 70–72). C3a and C5a are increased in the sputum and lavage of allergic asthma patients (44, 73, 74) and are thought to be particularly important in the acute phase of the disease (58). In particular, C3aR1 seems to be required to drive the Th2 axis (75) even in direct response to IL-33 (41). However, the functional links between complement anaphylatoxins, IL-33 and type II inflammation had not been fully elucidated. In our study, we demonstrated that primary hMCs respond differentially to anaphylatoxin stimulation, therefore suggesting that the activities of C3a and C5a on human MC activation are distinct, in keeping with the proposed role for C5a in established inflammatory environments. Furthermore, IL-33 is a crucial regulator of MC responses to complement anaphylatoxins suggesting a novel mechanism by which it might contribute to a MC driven feed forward amplification of Th2 specific immune responses leading to disease (41, 76, 77). This novel cross-regulation may explain exacerbated complement- and MC-dependent Th2 responses and thus provides an additional rationale for targeting anti-IL33 therapeutically in allergic diseases.

## Data Availability Statement

The raw data supporting the conclusions of this article will be made available by the authors, without undue reservation.

## Ethics Statement

The studies involving human participants were reviewed and approved by University of Manchester research ethics committee. The patients/participants provided their written informed consent to participate in this study.

## Author Contributions

PW, LR, and SB-P participated in the research design. PW, RB, KG-R, GW, and GS conducted the experiments. PW, RB, and KG-R performed data analysis. RS and AM provided lung tissues. PW, LR, and SB-P contributed to the writing of the manuscript, and all authors reviewed the final version. All authors contributed to the article and approved the submitted version.

## Funding

The research was supported by a GSK research grant; Manchester Allergy and Respiratory Thoracic Surgery (ManARTS) Biobank at University Hospital of South Manchester NHS Foundation Trust which is supported by the NIHR Manchester Biomedical Research Centre; PW was supported by a GSK research grant; RB was supported by a CRUK research grant; KG-R was supported by a CONACyT fellowship; LR is an employee at GSK; SB-P is supported by the University of Manchester and by an MRC research grant MR/S036954/1.

## Conflict of Interest

LR is an employee at GSK.

The remaining authors declare that the research was conducted in the absence of any commercial or financial relationships that could be construed as a potential conflict of interest.
