# Supplementary material for: Interleukin-33 Amplifies Human Mast Cell Activities Induced by Complement Anaphylatoxins
Source: Front Immunol. 2021 Feb 1;11:615236. doi: 10.3389/fimmu.2020.615236 (PMC7882629; doi:10.3389/fimmu.2020.615236)
Supplement: Supplementary file 1 [file DataSheet_1.docx]

Supplementary Material

# Supplementary Methods

## Generation of human peripheral blood derived mast cells

Peripheral blood derived mast cells were generated as previously described (E1, E2). Briefly, leukocyte cones were sourced from National Health Service Blood and Transplant blood bank (Manchester, UK) from anonymous healthy volunteers who had given consent for their donation to be used for research purposes, according to a protocol approved by the University of Manchester Research Ethics Committee (UREC ref 2018-2696-5711). PBMCs were isolated by ficoll-paque density gradient centrifugation and CD117^+^ progenitor cells were separated by positive magnetic selection using Macs™ CD117^+^ human microbead kit (Miltenyi Biotec, Bisley, UK) according to the manufacturer’s instructions. Isolated cells were grown in media containing human IL-3 (10 ng.ml^-1^), human IL-6 (50 ng.ml^-1^), and human stem cell factor (100 ng.ml^-1^) for 28 days before medium was progressively changed to that containing no IL-3. All media was supplemented with 1x insulin transferrin selenium solution (final concentration of 10 µg.ml^-1^, 5.5 µg.ml^-1^, 6.7 pg.ml^-1^ respectively), 100 U.ml^-1^ penicillin, 100 μg.ml^-1^ streptomycin and 0.5% BSA. Cells were tested for maturity at 8-10 weeks by flow cytometric analysis of CD117 and FcεRI expression and degranulation in response to IgE/αIgE. All cells had degranulation of greater than 60% at maturity with donor average of approximately 75%.

## Isolation of human lung tissue mast cells

Human lung tissue was obtained from three separate individuals without chronic lung disease undergoing thoracic surgery at Manchester University NHS foundation trust under the auspices of the Manchester Allergy, Respiratory and Thoracic Surgery (ManARTS) Biobank. The biobank was approved by the National Research Ethics Service Committee (ref; 15/NW/0409). Tissue was dissected by a pathologist to obtain healthy regions (>6 cm) distal to tumors. Human lung mast cells (hLMC) were obtained from this tissue according to a WEMP protocol previously described (E3). As described in the protocol, lung tissue was washed and dissected into small pieces before digestion with collagenase II in pre-warmed enzyme buffer (RPMI supplemented with 15 mM HEPES). The protocol was modified with increased enzymatic digestion time of 4 hours and addition DNAse I for only the final 45 minutes. Digestion was carried out with only gentle agitation in an incubator at 37°C. Digestion as halted with ice cold media (RPMI, 10% FCS, 100 U.ml^-1^ penicillin, 100 μg.ml^-1^ streptomycin). Mechanical disruption was carried out with scissors and 50 ml syringe before filtration, as described (E3). Cells were isolated from the collected filtrate using an Optiprep™ density gradient where cells were resuspended in a cushion with a density of 1.15 g.ml^-1^, overlayed with density of 1.067 g.ml^-1^, then overlayed with PBS before centrifugation at 1000 x g for 30 mins. Remaining red blood cells were lysed in ACK lysis buffer for 5 minutes and mast cells maintained in IMDM supplemented with human stem cell factor (100 ng.ml^-1^), 1x insulin transferrin selenium solution (final concentration of 10 µg.ml^-1^, 5.5 µg.ml^-1^, 6.7 pg.ml^-1^ respectively), 100 U.ml^-1^ penicillin, 100 μg.ml^-1^ streptomycin and 0.5% BSA, for up to 7 days.

## Flow cytometric analysis of marker expression and degranulation

For degranulation assays with IgE/αIgE complexes, cells were coated overnight with 1µg.ml^-1^ of human IgE (Merck, Watford, UK) washed in PBS and activated with media containing 1µg.ml^-1^goat anti-human IgE (Insight Biotechnology, Wembley, UK) for 1 hour before flow cytometric analysis as below. All other ligands were added for 1 hour or times otherwise indicated at concentrations indicated. Cells which had been washed in FACS buffer (PBS, 2% FBS, 200 μM EDTA) were incubated with Fc receptor blocking reagent prior to the addition of antibodies specific to C3aR1 (clone hC3aRZ8), C5aR1 (clone S5/1), C5aR2 (clone 1D9-M12), CD46 (clone TRA-2-10), CD55 (clone JS11), CD59 (clone P282), CD63 (clone H5C6), CD107a (clone H4A3), CD117(clone A3C6E2), FcεRI (clone AER-37), CD45 (clone 2D1), haematopoietic lineage cocktail (clones RPA-2.10, OKT3, 61D3, CB16, HIB19, TULY56, HIR2) or isotype control antibodies. Alternatively, fluorescence minus one controls (FMOs) were used. Isotype controls were used in initial experiments to check for non-specific, non-epitope driven binding, particularly when single antibody staining was performed. Where no non-epitope driven binding was observed, isotype controls were removed for subsequent multi-colour assays. Where anomalous results were observed for a specific fluorophore label of a single isotype that was not observed for the same isotype with a different label, we discounted this isotype in favour of FMO control. Cells were washed in PBS prior to incubation with live/dead™ reagent, again washed in PBS and analysed. For intracellular staining, cells were washed in PBS prior to incubation with live/dead™ reagent and washed in PBS before fixation with 4% formaldehyde solution. Cells were washed and incubated with Fc blocking reagent and antibodies (as above) in 1x permeabilisation buffer. Cells were washed and resuspended in FACS buffer before analysis. Cells were analysed on LSR-II, LSRFostessa and FACSymphony A5 instruments, and FlowJo software.

## Flow cytometric quantification of marker expression using Quantibrite™ beads

Beads and cells were quantified in the PE channel for each experiment. Each bead peak (low (A), medium-low (B), medium-high (C) and high (D)) was identified and a geometric (geo) mean fluorescence (GMFI) obtained. The defined number of PE molecules per bead was supplied by the manufacturer. Log mean fluorescence was plotted against the log PE molecules per bead. The PE molecules per cell was calculated from the fluorescence value by regression analysis using the equation of the line (y=mx + c). The number of bound antibodies per cell was calculated from the specific PE molecules by multiplying by the manufacturer supplied ratios of fluorescent molecules per antibody (F:P). Specific values were obtained by subtracting the appropriate isotype control value from the target antibody value.

## Flow cytometric analysis of ERK1/2 phosphorylation

Phosphoprotein analysis was carried out according to an established protocol (E4). Briefly, cells were washed in PBS and incubated for 10 mins with live/dead™ reagent prior to washing and resuspension in culture medium. Cells were activated as specified with complement anaphylatoxins C3a and C5a (50 nM) and fixed at specified time points (0-45 mins) by addition of formaldehyde to a final concentration of 1.6%. Cells were washed and permeabilised in methanol before incubation with phospho-ERK specific antibody (clone MILAN8R) and subject to flow cytometric analysis as described above.

## Analysis of gene expression by real-time PCR

Cells were pre-incubated where stated for 24 hours in control media, IL-33 (50 ng.ml^-1^) or IFNγ (50 ng.ml^-1^) and then washed in media and activated (where stated) for 8 hours with C3a (50 nM), C5a (50 nM) or CIA23187 (100 nM). RNA was isolated from cell pellets using RNeasy Micro Kit (Qiagen, Manchester, UK) and reverse transcribed to cDNA using Tetro™ cDNA synthesis kit (Bioline, Memphis, USA). Complimentary DNA was subject to quantitative PCR using fast SYBR™ green master mix and primers specific to CD46, C3aR1, C5aR1, C5aR2, C3, C4, C5, PPIA, H3F3B and 18S. Samples were run in triplicate using QuantStudio 12k Flex thermocycler and software v1.2.2. Relative quantification was performed using means of triplicate wells where the “housekeeping” was a mean of cycle threshold (CT) values from 3 genes (PPIA, H3F3B & 18S). Where given, 1/ΔCT = 1/(housekeeping-target) where housekeeping is a mean of CT for 3 genes; PPIA, H3F3B & 18S. Primer sequences, reaction volumes and cycle conditions are given in Supplementary table 1.

## Ratiometric measurement of mast cell calcium flux

Optically clear black walled 96-well culture plates were coated with poly-L-lysine (0.01% w/v in 0.1M Borate buffer pH 8.5) for 30 mins at 37°C and washed with PBS. Cells were plated at a concentration of 30,000 cells per well in culture medium and allowed to adhere for 1 hour at 37°C, 5%CO2. Cells were washed gently in calcium buffer (125 mM NaCl, 5.4 mM KCl, 16.2 mM NaHCO3, 0.8 mM MgCl2, 5.5 mM D-glucose, 20 mM HEPES, 1mM NaH2PO4, 1.8 mM CaCl2) with the addition of 0.1% BSA. Cells were loaded with 2 μM Fura-2 AM, in calcium buffer supplemented 0.02% w/v pluronic F-127, 2.75 mM probenecid and 1% BSA for 1 hour before washing in wash buffer. Assay buffer (calcium buffer supplemented 2.75 mM probenecid, 0.1% BSA) with was added. Agonists were diluted to achieve final concentrations specified in assay buffer. For experiments where calcium was absent, all buffers and chemicals were made with calcium buffer containing no CaCl2 but 2.6 mM MgCl2. Experiments were performed using a FlexStation 3 plate reader and SoftMax Pro software v5.4. Dual fluorescent excitation wavelengths were 340 and 380 nm respectively with emission wavelength of 510 nm and a cut-off of 495 nm. Readings were continuous for 450 seconds with a reading interval every 4 seconds. Ratiometric 340/380 measurements were calculated by the software with an automatic baseline based on three initial readings. Agonists were added at 30 seconds and change in fluorescence (ΔF) calculated from an average of 5 readings after the highest value, minus 5 readings directly before agonist addition. Time to peak (ΔT) was taken from the time point of the peak reading. EC50 values were determined using 4-parameter variable slope model in Graphpad Prism software v7.00.

## Measurement of chemokine and anaphylatoxin secretion

Measurement of chemokine or anaphylatoxin secretion was performed using cytometric bead array (CBA) human anaphylatoxin kit, or CBA flex sets specific to CXCL8/IL-8 or CCL2/MCP-1 (BD Biosciences) according to the manufacturer’s instructions and analysed using FACSVerse flow cytometer and FCAP Array™ software v 3.0. Briefly, collected cell-free supernatants or standards were incubated in low bind 96-well plates with pre-mixed, differentially fluorescent specific antibody-bead conjugates and PE-labelled detection reagent. After washing, samples were analysed and beads separated by differential fluorescence in the detection ranges of 660/10 nm and 783/56 nm. Phycoerythrin (PE) fluorescence was correlated with the concentration of chemokine or anapylatoxin by regression analysis using a standard curve. Values greater than two standard deviations from the mean were excluded from analysis.

## Additivity Models of Calcium Flux

Dose additivity models were created by converting change in fluorescence measurements at given C3a or C5a concentrations to percentage of maximum response, where 50nM of both C3a and C5a were 100%. The doses of C3a and C5a required to elicit a given percentage response (eg 20%, 40% of max *etc*) were interpolated from dose response curves. The *x,y* dose coordinates for a given % response (r) “topographic contour” were plotted as r([C3a],[C5a]) where the intersection of the x and y axis were set to r([C3a],0) and r(0,[C5a]). A perfect additive dose model would produce a straight line between these points, where reduction in [C3a] is perfectly compensated by increase in [C5a] or *vice versa*, therefore maintaining the % response at equally additive concentrations. Deviation from the line indicates synergism or antagonism at given % response and concentration.

Response additivity was analysed with a background reduced response (r’) equation:

$$r^{'}\left( \left[ C3a \right],\left[ C5a \right] \right)=r^{'}\left( \left[ C3a \right],0 \right)+r^{'}\left( 0,\left[ C5a \right] \right)-r^{'}\left( \left[ C3a \right],0 \right)r^{'}\left( 0,\left[ C5a \right] \right).$$

Where:

$$r^{'}=r\left( \left[ C3a50nM \right],[C5a50nM] \right)-r\left( 0,0 \right).$$

Expected response results from this model were compared to observed values using the equation:

$r^{'}\left( observed \right)-r'(expected)$.

Where a positive and negative values indicate percentage synergism or antagonism.

## References

Bahri R, Custovic A, Korosec P, Tsoumani M, Barron M, Wu J, Sayers R, Weimann A, Ruiz-Garcia M, Patel N, et al. Mast cell activation test in the diagnosis of allergic disease and anaphylaxis. J Allergy Clin Immunol (2018) 142:485-496.e16. doi:10.1016/J.JACI.2018.01.043

Bahri R, Bulfone-Paus S. “Mast cell activation test (MAT),” in Methods in Molecular Biology (Humana Press Inc.), 227–238. doi:10.1007/978-1-0716-0696-4_1

Ravindran A, Rönnberg E, Dahlin JS, Mazzurana L, Säfholm J, Orre A-C, Al-Ameri M, Peachell P, Adner M, Dahlén S-E, et al. An Optimized Protocol for the Isolation and Functional Analysis of Human Lung Mast Cells. Front Immunol (2018) 9:2193. doi:10.3389/fimmu.2018.02193

Krutzik PO, Nolan GP. Intracellular phospho-protein staining techniques for flow cytometry: Monitoring single cell signaling events. Cytometry (2003) 55A:61–70. doi:10.1002/cyto.a.10072

# Supplementary Figures and Tables

## Supplementary Tables

### Supplementary Table 1. Primer Sequences used in qPCR experiments.

| 18S forward | CCTGCGGCTTAATTTGACTC |
| --- | --- |
| 18S reverse | ATGCCAGAGTCTCGTTCGTT |
| H3F3B forward | GTTTGGTCGTTCGTTGGGC |
| H3F3B reverse | CTTTCACCCAACGCCGAAGT |
| PPIA forward | GGCAAATGCTGGACCAAAC |
| PPIA reverse | CATTCCTGGACCCAAAACG |
| C3aR1 forward | CCATTAGCCTGGATCGCTGT |
| C3aR1 reverse | ATCACAAAAGCCACCACCCA |
| C5aR1 forward | GCCCAGGAGACCAGAACAT |
| C5aR1 reverse | GGGTGTTGAGGTCCAGGGTA |
| C5aR2 forward | CAGTGTGTGGTGGACTACGG |
| C5aR2 reverse | AAAAACCCCACCACAATGGC |
| C3 forward | CGCCCGTGATACACCAAGAA |
| C3 reverse | TGAGAACAAAGGCCGTGAGG |
| C4 forward | CGTCACCCTCCTGAGTGGATT |
| C4 reverse | GGTGCCCCGTAAAACACAGA |
| C5 forward | GCCAAGAAGAACGCTGCAAA |
| C5 reverse | TCGCTGCTCACAGGTTTCAT |
| CD46 forward | GCCTACTTACAAGCCTCCAG |
| CD46 reverse | AGTGGCATATTCAGCTCCACC |

### Supplementary Table 2. PCR cycle conditions used for qPCR experiments

| **Hold Stage** | 95°C | 20 sec |
| --- | --- | --- |
| **40 cycles** | 95°C | 1 sec |
|  | 60°C | 20 sec |
| **Melt Curve** | 95°C | 15 sec |
|  | 60°C | 60 sec |
|  | Ramp | 0.05°C/sec |
|  | 95°C | 15 sec |

### Supplementary Table 3. Reaction volumes used on qPCR experiments.

| **Target** | **C3, CD46** | **C4** | **C5aR1, C3aR1, C5, PPIA** | **C5aR2, 18S, H3F3B** |
| --- | --- | --- | --- | --- |
| **SYBR™green** | 5μl | 5μl | 5μl | 5μl |
| **10μM F primer** | 0.125μl | 0.125μl | 0.25μl | 0.05μl |
| **10μM R primer** | 0.125μl | 0.125μl | 0.25μl | 0.125μl |
| **MgCl_2_ (25mM)** | - | 0.05μl | - | - |
| **H_2_O** | 4.25μl | 4.2μl | 4μl | 4.325μl |
| **cDNA** | 0.5μl | 0.5μl | 0.5μl | 0.5μl |
| **TOTAL** | 10μl | 10μl | 10μl | 10μl |

### Supplementary Table 4. Summary Statistics

**2.1.4.1** **Table 4A.** Summary Statistics for Figure 1.

| Figure | Panel | N | Test | Means | Medians | SDs | SEMs | Mean or Median of Differences | 95% CI | Summary  (p-value) |
| --- | --- | --- | --- | --- | --- | --- | --- | --- | --- | --- |
| 1 | A1 | 6, 6 | Wilcoxon matched-pairs signed rank test | 59.85, 988.1 | 63.55, 810.5 | 23.12, 353.8 | 9.437, 144.5 | 759.4 | 667.9 to 1446 (96.88% CI) | * (0.0313) |
|  | A2 | 6, 6 | Wilcoxon matched-pairs signed rank test | 28.93, 417.2 | 23.25, 216.5 | 20.17, 385.7 | 8.233, 157.5 | 172.9 | 87.2 to 986.4 (96.88% CI) | * (0.0313) |
|  | A3 | 6, 6 | Paired t-test | 63.08, 79.99 | 56.70, 82.63 | 26.35, 24.33 | 10.76, 9.931 | 16.91 | 0.4424 to 33.37 | * (0.0460) |
|  | B1 | 6, 6 | Paired t-test | 177.0  1257 | 149.9  1345 | 151.8  391.1 | 61.98  159.7 | 1080 | 718.8 to 1441 | *** (0.0006) |
|  | B2 | 6, 6 | Paired t-test | 47.27  595.2 | 47.50  566.8 | 13.99  135.9 | 5.710  55.47 | 547.9 | 416.3 to 679.5 | *** (0.0001) |
|  | B3 | 6, 6 | Paired t-test | 245.7  1072 | 272.5  1004 | 85.02  412.7 | 34.71  168.5 | 826.8 | 372.2 to 1281 | ** (0.0055) |
|  | C1 | 6,  6,  6 | One-way ANOVA (Dunnett’s multiple comparison post-test) | 1,  0.3285,  0.8210 | 1,  0.2866,  0.8727 | 0,  0.1406,  0.1519 | 0,  0.05740,  0.06200 | 0.6715,  0.1790 | 0.4975 to 0.8455,  -0.0089 to 0.3669 | *** (0.0002)  n.s. (0.0588) |
|  | C2 | 6,  6,  6 | One-way ANOVA (Dunnett’s multiple comparison post-test) | 1,  0.6863,  0.3659 | 1,  0.6747,  0.5444 | 0,  0.3772,  0.6099 | 0,  0.1540,  0.2490 | 0.3137,  0.6341 | -0.1529 to 0.7804,  -0.1204 to 1.389 | n.s.  (0.1615)  n.s.  (0.0874) |
|  | C3 | 6,  6,  6 | One-way ANOVA (Dunnett’s multiple comparison post-test) | 1,  0.7931,  0.9215 | 1,  0.9171, 0.9950 | 0,  0.5817,  0.3058 | 0,  0.2375, 0.1248 | 0.2069,  0.0785 | -0.5128 to 0.9266,  -0.2998 to 0.4568 | n.s.  (0.6196)  n.s.  (0.7663) |
|  | D1 | 3,  3,  3 | One-way ANOVA (Dunnett’s multiple comparison post-test) | 1,  0.3746 0.3354 | 1,  0.3737 0.3170 | 0,  0.06624    0.03689 | 0,  0.03824 0.02130 | 0.6254  0.6646 | 0.4182 to 0.8326  0.5492 to 0.7800 | ** (0.0058)  ** (0.0016) |
|  | D2 | 3,  3,  3 | One-way ANOVA (Dunnett’s multiple comparison post-test) | 1,  0.5308 0.3072 | 1,  0.5225 0.3128 | 0,  0.1468  0.07087 | 0,  0.08475 0.04092 | 0.4692  0.6928 | 0.0100 to 0.9284  0.4712 to 0.9145 | *  (0.0480)  **  (0.0054) |
|  | D3 | 3,  3,  3 | One-way ANOVA (Dunnett’s multiple comparison post-test) | 1,  0.9380 1.264 | 1,  0.9649 1.454 | 0,  0.1109  0.4207 | 0,  0.06404 0.2429 | 0.03506  -0.4544 | -0.3119 to 0.3820  -1.770 to 0.8616 | n.s.  (0.8243)  n.s.  (0.3013) |

**2.1.4.2** **Table 4B.** Summary statistics for flow cytometric population comparisons for individual donors in Figure 1A.

| Figure | Panel | N | Test | Means | Value of T(X)  Summary of K-S Probability of Different Populations  (% positive cells by SE Dymax) |
| --- | --- | --- | --- | --- | --- |
| 1 | A1a  A1b  A1c  A1d  A1e  A1f | 1  1  1  1  1  1 | ChiSquare T(X),  SE Dymax % Positive  FMO/Isotype vs Specific Antibody | 24.8, 831  77.6, 1523.5  77.5, 790  46.6, 718.5  49.6, 717.5  83, 1348 | 1175.3785, >99.9% (99.8894%+)  807.9458, >99.9% (99.9321%+)  530.5576, >99.9% (99.6180%+)  1321.4509, >99.9% (98.8399%+)  1303.6958, >99.9% (99.8955%+)  892.5426, >99.9% (99.9417%+) |
|  | A2a  A2b  A2c  A2d  A2e  A2f | 1  1  1  1  1  1 | ChiSquare T(X),  SE Dymax % Positive  FMO/Isotype vs Specific Antibody | 15.1, 126  29.9, 165.5  48.3, 135.5  16.6, 1003  6.36, 805.5  57.3, 267.5 | 463.4886, >99.9% (79.9804%+)  247.6427, >99.9% (75.4257%+)  560, >99.9% (5.0889%+)%+))  130.5742, >99.9% (70.0542%+)  549.4885, >99.9% (99.1071%+)  1514.6862, >99.9% (97.9705%+)  417.2410, >99.9% (83.1929 %+) |
|  | A3a  A3b  A3c  A3d  A3e  A3f | 1  1  1  1  1  1 | ChiSquare T(X),  SE Dymax % Positive  FMO/Isotype vs Specific Antibody | 82.4, 87.05  105, 112  63.1, 78.2  50.3, 97.2  37.3, 45.65  40.4, 59.85 | 0.0, between 98-99% (5.8496%+)  3.2560, >99.9% (5.0889%+)  21.1713, >99.9% (34.4732%+)  38.2709, >99.9% (30.8385%+)  42.7865, >99.9% (12.2280%+)  61.1258, >99.9% (40.6052%+) |
|  | B1a  B1b  B1c  B1d  B1e  B1f | 1  1  1  1  1  1 | ChiSquare T(X),  SE Dymax % Positive  FMO/Isotype vs Specific Antibody | 30, 562  69.7, 1099  38.4, 1619  313, 1271  381, 1572  230, 1419.5 | 904.7286, >99.9% (99.6140%+)  754.1315, >99.9% (99.6811%+)  527.2406, >99.9% (99.9799%+)  14.0251, >99.9% (99.7506%+)  1206.6776, >99.9% (99.8421%+)  804.7007, >99.9% (99.2820%+) |
|  | B2a  B2b  B2c  B2d  B2e  B2f | 1  1  1  1  1  1 | ChiSquare T(X),  SE Dymax % Positive  FMO/Isotype vs Specific Antibody | 41.5, 522.5  53.5, 492.5  27.9, 436  62.2, 746.5  61.5, 762.5  37, 611 | 756.6357, >99.9% (99.8970%+)  1250.1537, >99.9% (99.8573%+)  434.2547, >99.9% (99.8350%+)  1021.8573, >99.9% (99.2226%+)  971.9320, >99.9% (99.5948%+)  487.1220, >99.9% (99.0922%+) |
|  | B3a  B3b  B3c  B3d  B3e  B3f | 1  1  1  1  1  1 | ChiSquare T(X),  SE Dymax % Positive  FMO/Isotype vs Specific Antibody | 147, 607.5  283, 1209  141, 1778  273, 832  272, 843  358, 1165 | 517.6078, >99.9% (96.2040%+)  941.5234, >99.9% (97.9675%+)  335.4880, >99.9% (98.9128%+)  1094.6644, >99.9% (99.0074%+)  1040.4355, >99.9% (99.3831%+)  255.7352, >99.9% (94.0976%+) |

**2.1.4.3 Table 4C.** Summary statistics for Figure 2.

| Figure | Panel | N | Test | Means | Medians | SDs | SEMs | Mean or Median of Differences | 95% CI | Summary  (p-value) |
| --- | --- | --- | --- | --- | --- | --- | --- | --- | --- | --- |
| 2 | A1 | 6,  6, 6,  6 | One-way ANOVA (Dunnett’s multiple comparison post-test) | 0.3611,  0.3338,  0.1309,  0.1366 | 0.3225  0.3490  0.1472  0.0753 | 0.1357  0.1900  0.0481  0.1074 | 0.05541  0.07757  0.01965  0.04386 | -0.02734  0.2029  0.1972 | -0.4290 to 0.3743  0.004472 to 0.4014  -0.1870 to 0.5813 | n.s.  (0.9913)  *  (0.0462)  n.s.  (0.3147) |
|  | A2 | 6,  6, 6,  6 | One-way ANOVA (Dunnett’s multiple comparison post-test) | 7423  14196  988.6  617.1 | 7593  14204  1128  410.2 | 868.9  7287  670.2  570.0 | 354.7  2975  273.6  232.7 | 6772  13207  13579 | -2370 to 15914  3431 to 22983  3503 to 23654 | n.s.  (0.1305)  *  (0.0159)  *  (0.0161) |
|  | B1  CD46 | 3,  3,  3 | One-way ANOVA (Dunnett’s multiple comparison post-test) | 1.000  0.6733  1.105 | 1.000  0.4545  1.348 | 0.000  0.5568  0.4335 | 0.000  0.3214  0.2503 | 0.3267  -0.1046 | -1.415 to 2.068  -1.461 to 1.251 | n.s.  (0.5856)  n.s.  (0.8884) |
|  | B2 C3aR1 | 3,  3,  3 | One-way ANOVA (Dunnett’s multiple comparison post-test) | 1.000  1.136  1.822 | 1.000  1.133  1.317 | 0.000  0.6848  1.415 | 0.000  0.3954  0.8172 | -0.1361  -0.8216 | -2.278 to 2.006  -5.249 to 3.606 | n.s.  (0.9210)  n.s.  (0.5906) |
|  | B3 C5aR1 | 3,  3,  3 | One-way ANOVA (Dunnett’s multiple comparison post-test) | 1.000  0.5460  1.083 | 1.000  0.6389  0.4038 | 0.000  0.3658  1.188 | 0.000  0.2112  0.6856 | 0.4540  -0.08260 | -0.6904 to 1.598  -3.797 to 3.632 | n.s.  (0.2472)  n.s.  (0.9894) |
|  | B4 C5aR2 | 3,  3,  3 | One-way ANOVA (Dunnett’s multiple comparison post-test) | 1.000  1.382  0.4969 | 1.000  1.295  0.3964 | 0.000  0.9566  0.4861 | 0.000  0.5523  0.2807 | -0.3816  0.5031 | -3.374 to 2.611  -1.018 to 2.024 | n.s.  (0.7490)  n.s.  (0.3191) |

**2.1.4.4. Table4D.** Summary statistics for Figure 3.

| Figure | Panel | N | Test | Means | Medians | SDs | SEMs | Mean or Median of Differences | 95% CI | Summary  (p-value) |
| --- | --- | --- | --- | --- | --- | --- | --- | --- | --- | --- |
| 3 | A1 | 7,  7,  7,  7 | One-way ANOVA (Dunnett’s multiple comparison post-test)  Vs Control  Vs C5a  ^#^ Sidak’s adjustment lowers α as follows:  ** p*0.05=0.0399  *** p*0.01=0.003  **** p*0.001=0.00079 | 0.7581,  70.41,  44.01,  7.218 | 0.9000 75.85 50.13 3.530 | 0.4257 19.94 31.87 7.483 | 0.1609 7.538 12.05 2.828 | -69.65  -43.25  -6.460  6.460  -63.19  -36.79 | -94.56 to  -44.74  -68.16 to  -18.34  -31.37 to 18.45  -2.208 to 15.13  -89.00 to  -37.38  -70.12 to  -3.452 | ****  (<0.0001)  ***  (0.0009)^#^  n.s.  (0.8453)  n.s.  (0.1364)  ***  (0.0007)^#^  *  (0.0338)^#^ |
|  | A2 | 7,  7,  7,  7 | One-way ANOVA (Dunnett’s multiple comparison post-test)  Vs Control | 0.8736,  73.58,  35.35,  6.109, | 0.7150, 70.25, 25.25,  2.240, | 0.594, 16.29, 31.74, 7.789, | 0.2246, 6.156, 12.00, 2.944, | -72.71  -34.48  -5.236 | -95.49 to  -49.93  -57.26 to  -11.70  -28.02 to 17.55 | ****  (<0.0001)  **  (0.0030)  n.s.  (0.8845) |
|  | B |  | N/A |  |  |  |  |  |  |  |
|  | C1 | 3,  3 | Wilcoxon matched-pairs signed rank test | 1282  14068 | 1045  14258 | 428.2  5909 | 247.2  3411 | 13213 | - | n.s.  (0.2500) |
|  | C2 | 3,  3 | Paired t-test | 412.3,  566.3 | 482.0,  492.0 | 206.5,  235.5 | 119.2,  135.9 | 154.0 | -389.3 to 697.3 | n.s.  (0.3469) |
|  | C3 | 3,  3 | Paired t-test | 301.0,  361.0 | 280.0,  336.0 | 39.89,  54.03 | 23.03,  31.19 | 60.00 | 20.25 to 99.75 | *  (0.0229) |
|  | D | 3,  3,  3,  3 | One-way ANOVA (Tukey’s multiple comparison post-test) | 0.1811,  90.50,  84.33,  19.50 | 0.03600,  92.50,  87.10,  24.80 | 0.2765,  9.657,  7.636,  10.69 | 0.1597,  5.575  4.408,  6.173 | Ctrl vs  B -84.15,  C -19.32,  D -90.32,  CIA vs  C 64.84,  D -6.167,  C3a vs  D -71.00 | -128.1 to  -52.56,  -114.0 to  -24.29,  -63.18 to 24.55,  -42.50 to 54.83,  -4.248 to 146.3,  -3.359 to 133.0 | **  (0.0093),  **  (0.0068),  n.s.  (0.2189),  n.s.  (0.8202),  n.s.  (0.0559)  n.s.  (0.0551), |
|  | E1 | 3,  3,  3 | One-way ANOVA (Dunnett’s multiple comparison post-test) | 1,  0.4497,  0.9723 | 1,  0.4890,  0.9581 | 0,  0.1144,  0.0689 | 0,  0.0660,  0.0398 | 0.5503,  0.0277 | 0.1926 to 0.9081,  -0.1877 to 0.2432 | *  (0.0218)  n.s.  (0.7456) |
|  | E2 | 3,  3,  3 | One-way ANOVA (Dunnett’s multiple comparison post-test) | 1,  1.922,  -2.878 | 1,  1.929, -1.607 | 0,  2.003, 4.544 | 0,  1.156, 2.623 | -0.9220  3.878 | -7.188 to 5.344  -10.34 to 18.09 | n.s.  (0.6933)  n.s.  (0.4057) |

**2.1.4.5 Table 4E.** Summary statistics for flow cytometric population comparisons for individual donors in Figure 3C.

| **Figure** | **Panel** | **N** | **Test** | **Means** | **Value of T(X)**  **Summary of K-S Probability of Different Populations**  **(% positive cells by SE Dymax)** |
| --- | --- | --- | --- | --- | --- |
| 3 | C1a  C1b  C1c | 1  1  1 | ChiSquare T(X),  K-S Probability %  (SE Dymax %Positive) | 1045, 14258  1776, 19879  1024, 8066 | 536.4662, >99.9% (99.9776%+)  422.1465, >99.9% (99.9836%+)  882.5538, >99.9% (99.8287%+ |
|  | C2a  C2b  C2c | 1  1  1 | ChiSquare T(X),  K-S Probability %  (SE Dymax %Positive) | 180,377  482,830  575,492 | 16.8300, >99.9% (78.7795%+)  666.1017, >99.9% (49.4565%+)  (-)85.3214, >99.9% (0.0%+) |
|  | C3a  C3b  C3c | 1  1  1 | ChiSquare T(X),  K-S Probability %  SE Dymax %Positive | 347, 423  280, 324  276, 336 | 105.7562, >99.9% (16.1715%+)  94.8570, >99.9% (28.0530%+)  153.2827, >99.9% (39.4057%+) |

**2.1.4.6 Table 4F.** Summary statistics for Figure 4.

| Figure | Panel | N | Test | Means | Medians | SDs | SEMs | Mean or Median of Differences | 95% CI | Summary  (p-value) |
| --- | --- | --- | --- | --- | --- | --- | --- | --- | --- | --- |
| 4 | C | 2,  2,  2,  2 | One-way ANOVA (Dunnett’s multiple comparison post-test)  Vs column D | 5.753,  27.20,  33.30,  52.25 | 5.753,  27.20,  33.30,  52.25 | 2.019 0.6364 3.818 10.89 | 1.428 0.4500 2.700 7.700 | 46.50  25.05  18.95 | -136.4 to 229.4  -120.2 to 170.3  -81.24 to 119.1 | n.s.  (0.1935)  n.s.  (0.2797)  n.s.  (0.2565) |
|  | D | 3,  3,  3,  3 | One-way ANOVA (Dunnett’s multiple comparison post-test)  Vs column D | 250.4 250.7 410.3 310.3 | 267.0 191.2 383.2 236.0 | 103.0 158.7 83.51 136.0 | 59.46 91.64 48.22 78.53 | 59.87  59.57  -100.0 | -245.0 to 364.7  -80.01 to 199.1  -309.4 to 109.3 | n.s.  (0.5974)  n.s.  (0.2268)  n.s.  (0.1890) |
|  | E | 2,  2,  2,  2 | One-way ANOVA (Dunnett’s multiple comparison post-test)  Vs column D | 5.753,  51.68,  37.65,  72.28 | 5.753,  51.68,  37.65,  72.28 | 2.019 8.238 3.677 1.450 | 1.428 5.825 2.600 1.025 | -45.92  -31.90  -66.52 | 17.38 to 115.7  -116.7 to 157.9  3.065 to 66.18 | *  (0.0370)  n.s.  (0.3178)  *  (0.0456) |
|  | F | 3,  3,  3,  3 | One-way ANOVA (Dunnett’s multiple comparison post-test)  Vs column D | 250.4 149.0 518.6 252.8 | 267.0 175.4 596.6 277.6 | 103.0 47.13 169.1 170.4 | 59.46 27.21 97.65 98.36 | 2.352  103.8  -265.8 | -946.9 to 951.6  -537.5 to 745.1  -565.4 to 33.83 | n.s.  (>0.9999)  n.s.  (0.6944)  n.s.  (0.0627) |

**2.1.4.7 Table 4G.** Summary Statistics for Figure 5.

| Figure | Panel | N | Test | Means | Medians | SDs | SEMs | Mean or Median of Differences | 95% CI | Summary  (p-value) |
| --- | --- | --- | --- | --- | --- | --- | --- | --- | --- | --- |
| 5 | A1 | 6, 6,  6,  6,  6,  6 | Two-way ANOVA (Sidak’s multiple comparison post-test)  Ctrl vs IL-33 | 0.9553,  1.9492,  51.397,  70.286  21.959,  31.166 | 0.876, 4.375  52.3,  88.9  18.95, 53.55 | 0.6096  1.333  17.393  11.283  14.121  17.805 | 0.249, 0.544  7.101, 4.606  5.765, 7.269 | -0.9939  -18.89  -9.206 | -7.211 to 5.223  -25.11 to -12.67  -15.42 to -2.988 | n.s.  (0.9597)  ****  (<0.0001)  **  (0.0052) |
|  | A2 | 6, 6,  6,  6,  6,  6 | Two-way ANOVA (Sidak’s multiple comparison post-test)  Ctrl vs IL-33 | 1.322, 1.682  48.578, 69.925  21.689, 30.553 | 1.533, 4.375  47.633, 90.1  19.7, 53.55 | 0.739, 1.404  20.754, 10.869  11.332, 17.404 | 0.302, 0.573  8.473, 4.437  4.626, 7.105 | -0.3606  -21.35  -8.864 | -9.351 to 8.630  -30.34 to -12.36  -17.86 to 0.1264 | n.s.  (0.9993)  ***  (0.0001)  n.s.  (0.0535) |
|  | B1 | 3,2  3,3  3,3 | Two-way ANOVA (Tukey’s multiple comparison post-test)  Ctrl/Ctrl (A),  IL-33/Ctrl (B)  Ctrl/C3a (C),  IL-33/C3a (D)  Ctrl/C5a, (E)  IL-33/C5a (F) | 4.25, 293.37  10.357, 713.39  9.177, 606.68 | 2.310, 293.370  9.810, 649.770  9.550, 634.525 | 5.0572, 2.305  2.771, 110.739  1.525, 49.132 | 2.920, 1.630  1.600, 63.935  0.880,  28.367 | Ctrl/Ctrl vs  B -289.1  C -6.107  D -709.1  E -4.927  F -602.4  IL-33/Ctrl vs  C 283.0  D -420.0  E 284.2  F -313.3  Ctrl/C3a vs  D -703.0  E 1.180  F -596.3  IL-33/C3a vs  E 704.2  F 106.7  Ctrl/C5a vs  F -597.5 | -464.5 to -113.7  -163.0 to 150.8  -866.0 to -552.3  -161.8 to 152.0  -759.3 to -445.5  107.6 to 458.4  -595.4 to -244.6  108.8 to 459.6  -488.7 to -137.9  -859.9 to -546.2  -155.7 to 158.1  -753.2 to -439.4  547.3 to 861.1  -50.17 to 263.6  -754.4 to -440.6 | **  (0.0011)  n.s.  (>0.9999)  ****  (<0.0001)  n.s.  (>0.9999)  ****  (<0.0001)  **  (0.0014)  ****  (<0.0001)  **  (0.0013)  ***  (0.0006)  ****  (<0.0001)  n.s.  (>0.9999)  ****  (<0.0001)  ****  (<0.0001)  n.s.  (0.3483)  ****  (<0.0001) |
|  | B2 | 3,2  3,3  3,3 | Two-way ANOVA (Tukey’s multiple comparison post-test)  Ctrl/Ctrl (A),  IL-33/Ctrl (B)  Ctrl/C3a (C),  IL-33/C3a (D)  Ctrl/C5a, (E)  IL-33/C5a (F) | 253.583, 570.875  361.377, 591.947  428.837,1348.11 | 254.98, 570.875  350.66, 552.5  438.81, 1346.34 | 11.091, 79.924  30.530, 74.131  29.178, 73.891 | 6.403, 56.515  17.626, 42.799  16.846, 42.661 | Ctrl/Ctrl vs  B -317.3  C -107.8  D -338.4  E -175.3  F -1095  IL-33/Ctrl vs  C 209.5  D -21.07  E 142.0  F -777.2  Ctrl/C3a vs  D -230.6  E -67.46  F -986.7  IL-33/C3a vs  E 163.1  F -756.2  Ctrl/C5a vs  F -919.3 | -485.5 to -149.1  -258.2 to 42.65  -488.8 to -187.9  -325.7 to -24.81  -1245 to -944.1  41.29 to 377.7  -189.3 to 147.1  -26.17 to 310.2  -945.4 to -609.0  -381.0 to -80.12  -217.9 to 82.99  -1137 to -836.3  12.66 to 313.6  -906.6 to -605.7  -1070 to -768.8 | ***  (0.0005)  n.s.  (0.2216)  ***  (0.0001)  *  (0.0203)  ****  (<0.0001)  *  (0.0131)  n.s.  (0.9977)  n.s.  (0.1156)  ****  (<0.0001)  **  (0.0029)  n.s.  (0.6549)  ****  (<0.0001)  *  (0.0315)  ****  (<0.0001)  ****  (<0.0001) |
|  | C1 | 3,3  3,  3  3,  3 | Two-way ANOVA (Sidak’s multiple comparison post-test)  Ctrl vs IL-33 | 0.112, 0.126  1.547, 1.811  0.986, 1.191 | 0.093, 0.065  1.458, 1.589  0.885, 1.159 | 0.094, 0.143  0.805, 0.851  0.298, 0.276 | 0.054, 0.082  0.465, 0.491  0.172, 0.159 | -0.01424  -0.2643  -0.2055 | -0.3583 to 0.3299  -0.6084 to 0.0798  -0.5496 to 0.1386 | n.s.  (0.9982)  n.s.  (0.1122)  n.s.  (0.2168) |
|  | C2 | 3,3  3,  3  3,  3 | Two-way ANOVA (Sidak’s multiple comparison post-test)  Ctrl vs IL-33 | 131.141, 125.026  74.924, 74.924  42.559, 26.655 | 106.329, 179.529  74.924, 74.924  40.033, 26.914 | 118.595, 100.246  0,  0  21.475, 1.870 | 68.471, 57.877  0,  0  12.399, 1.0795 | 6.115  0.000  15.90 | -115.9 to 128.1  -122.0 to 122.0  -106.1 to 137.9 | n.s.  (0.9968)  n.s.  (>0.9999)  n.s.  (0.9512) |
|  | D1 | 3,3,3 | One-way ANOVA (Dunnett’s multiple comparison post-test)  Vs Ctrl | 1.000 1.124 1.096 | 1.000 1.093 1.134 | 0.000 0.1297 0.2908 | 0.000 0.07491 0.1679 | -0.1237  -0.09583 | -0.5535 to 0.3062  -0.5257 to 0.3340 | n.s.  (0.6439)  n.s.  (0.7586) |
|  | D2 | 3,3,3 | One-way ANOVA (Dunnett’s multiple comparison post-test)  Vs Ctrl | 1.000 0.5775 0.9135 | 1.000 0.4940 0.9675 | 0.000 0.1749 0.1209 | 0.000 0.1010 0.06981 | 0.4225  0.08650 | 0.1355 to 0.7095  -0.2005 to 0.3735 | **  (0.0100)  n.s.  (0.6198) |
|  | D3 | 3,3,3 | One-way ANOVA (Dunnett’s multiple comparison post-test)  Vs Ctrl | 1.000  0.6040  0.8797 | 1.000  0.5820  0.8780 | 0.000  0.1146    0.2665 | 0.000 0.06616 0.1539 | 0.3960  0.1203 | 0.00447 to 0.7875  -0.2712 to 0.5119 | *  (0.0479)  n.s.  (0.6096) |
|  | D4 | 3,3,3 | One-way ANOVA (Dunnett’s multiple comparison post-test) | 1.000  0.8067  0.8278 | 1.000  0.7590  0.6920 | 0.000  0.1541  0.4833 | 0.000  0.08899  0.2790 | 0.1933  0.1722 | -0.2888 to 0.6755  -1.340 to 1.684 | n.s.  (0.2433)  n.s.  (0.7880) |
|  | E1 | 4,  4,  4 | Two-way ANOVA (Tukey’s multiple comparison post-test) | 2.597  117.126  3.613 | 2.705  108.295  2.767 | 1.251  44.251  2.345 | 0.625  22.125  1.172 | Ctrl vs IL-33  -114.5  Ctrl vs IFNg  -1.016  IL-33 vs IFNg  113.5 | -177.0 to -52.03  -63.52 to 61.49  52.88 to 174.1 | ***  (0.0004)  n.s.  (0.9991)  ***  (0.0003) |
|  | E2 | 3,3,3  3,3,3  3,3,3  3,3,3 | Two-way ANOVA (Tukey’s multiple comparison post-test) | Ctrl  1  0.870  13.983  C3a  0.861  0.786  3.915  C5a  1.153  4.191  6.784  CIA  4.317  3.851  8.923 | Ctrl  1  0.880045  2.762042  C3a  0.405357  0.481712  2.654132  C5a  0.877396  1.015723  5.670016  CIA  1.855575  1.162427  11.74828 | Ctrl  0  0.172  21.473  C3a  0.805  0.931  2.460  C5a  0.906  5.764  6.235  CIA  4.730  5.005  5.974 | Ctrl  0  0.099  12.397  C3a  0.465  0.538  1.420  C5a  0.523  3.328  3.600  CIA  2.731  2.890  3.449 | Ctrl  Ctrl vs IL-33  0.1301  Ctrl vs IFNg  -12.98  IL-33 vs IFNg  -13.11  C3a  Ctrl vs IL-33  0.07460  Ctrl vs IFNg  -3.054  IL-33 vs IFNg  -3.129  C5a  Ctrl vs IL-33  -3.038  Ctrl vs IFNg  -5.630  IL-33 vs IFNg -2.593  CIA23187  Ctrl vs IL-33 0.4660  Ctrl vs IFNg -4.605  IL-33 vs IFNg -5.071 | -12.72 to 12.98  -25.83 to -0.1377  -25.96 to -0.2678  -12.77 to 12.92  -15.90 to 9.791  -15.97 to 9.717  -15.88 to 9.808  -18.48 to 7.215  -15.44 to 10.25  -12.38 to 13.31  -17.45 to 8.240  -17.92 to 7.774 | n.s.  (0.9996)  *  (0.0475)  *  (0.0453)  n.s.  (0.9999)  n.s.  (0.8044)  n.s.  (0.7960)  n.s.  (0.8063)  n.s.  (0.4925)  n.s.  (0.8541)  n.s.  (0.9949)  n.s.  (0.6167)  n.s.  (0.5593) |
|  | E3 | 3,3,3  3,3,3  3,3,3  3,3,3 | Two-way ANOVA (Tukey’s multiple comparison post-test) | Ctrl  1  1.428  1.384  C3a  0.321  0.326  0.441  C5a  0.550  0.208  0.085  CIA  0.289  0.215  0.927 | Ctrl  1  0.514  0.830  C3a  0.339  0.396  0.331  C5a  0.479  0.209  0.094  CIA  0.093  0.111  1.155 | Ctrl  0  1.698  1.689  C3a  0.199  0.270  0.230  C5a  0.311  0.196  0.056  CIA  0.412  0.275  0.699 | Ctrl  0  0.981  0.975  C3a  0.115  0.156  0.133  C5a  0.179  0.113  0.032  CIA  0.238  0.159  0.403 | Ctrl  Ctrl vs IL-33  -0.4285  Ctrl vs IFNg  -0.3843  IL-33 vs IFNg  0.04412  C3a  Ctrl vs IL-33  -0.004911  Ctrl vs IFNg  -0.1201  IL-33 vs IFNg  -0.1152  C5a  Ctrl vs IL-33  0.3420  Ctrl vs IFNg  0.4650  IL-33 vs IFNg  0.1229  CIA  Ctrl vs IL-33  0.07364  Ctrl vs IFNg  -0.6385  IL-33 vs IFNg  -0.7121 | -2.202 to 1.345  -2.158 to 1.389  -1.729 to 1.818  -1.778 to 1.769  -1.893 to 1.653  -1.889 to 1.658  -1.431 to 2.115  -1.308 to 2.238  -1.651 to 1.896  -1.700 to 1.847  -2.412 to 1.135  -2.486 to 1.061 | n.s.  (0.7989)  n.s.  (0.8341)  n.s.  (0.9976)      n.s.  (>0.9999)  n.s.  (0.9822)  n.s.  (0.9836)      n.s.  (0.8658)  n.s.  (0.7683)  n.s.  (0.9814)      n.s.  (0.9933)  n.s.  (0.6143)  n.s.  (0.5488) |

**2.1.4.8 Table 4H.** Summary Statistics for Figure 6.

| Figure | Panel | N | Test | Means | Medians | SDs | SEMs | Mean or Median of Differences | 95% CI | Summary  (p-value) |
| --- | --- | --- | --- | --- | --- | --- | --- | --- | --- | --- |
| 6 | C | 7,  7,  7,  7,  7,  7,  7,  7 | Two-way ANOVA (Sidak’s multiple comparison post-test)  Ctrl vs IL-33 at each time point | 1,  0.939  1.615, 1.644  4.614, 6.833  6.498, 9.627  6.526, 7.891  5.305, 6.393  2.413, 3.449  1.676, 1.704 | 1,  0.919  1.328, 1.643  4.683, 7.288  7.526, 10.457  6.719, 7.181  5.567, 5.197  1.964, 2.937  1.866, 1.752 | 0,  0.113  0.676, 0.411  2.497, 3.402  3.165, 5.546  3.759, 4.965  3.145, 4.371  1.478, 1.962  0.490, 0.287 | 0,  0.043  0.255, 0.155  0.944, 1.286  1.196, 2.096  1.421, 1.877  1.189, 1.652  0.559, 0.742  0.185, 0.108 | 0.06121  -0.02903  -2.219  -3.130  -1.365  -1.088  -1.036  -0.02820 | -1.479 to 1.602  -1.569 to 1.511  -3.759 to -0.6784  -4.670 to -1.589  -2.906 to 0.1750  -2.628 to 0.4528  -2.577 to 0.5043  -1.569 to 1.512 | n.s.  (>0.9999)  n.s.  (>0.9999)  **  (0.0013)  ****  (<0.0001)  n.s.  (0.1118)  n.s.  (0.3313)  n.s.  (0.3919)  n.s.  (>0.9999) |
|  | E | 7,  7,  7,  7,  7,  7,  7,  7 | Two-way ANOVA (Sidak’s multiple comparison post-test)  Ctrl vs IL-33 at each time point | 1,  1.064  1.247, 1.434  3.018, 3.865  4.573, 5.561  5.507, 7.485  6.512, 9.537  4.971, 9.957  2.809, 5.112 | 1,  1.035  1.239, 1.211  2.740, 4.002  4.072, 6.167  4.051, 5.401  4.318, 4.404  4.428, 9.982  3.316, 3.309 | 0,  0.206  0.420, 0.656  1.607, 2.287  2.833, 3.348  4.118, 7.128  6.060, 9.970  3.558, 7.860  1.307, 3.983 | 0,  0.078  0.159, 0.248  0.607, 0.865  1.071, 1.265  1.557, 2.694  2.291, 3.768  1.345, 2.971  0.494, 1.506 | -0.06372  -0.1867  -0.8467  -0.9882  -1.979  -3.025  -4.986  -2.302 | -2.536 to 2.409  -2.659 to 2.286  -3.319 to 1.626  -3.461 to 1.484  -4.451 to 0.4938  -5.497 to -0.5524  -7.458 to -2.513  -4.775 to 0.1700 | n.s.  (>0.9999)  n.s.  (>0.9999)  n.s.  (0.9600)  n.s.  (0.9079)  n.s.  (0.1944)  **  0.0086  ****  (<0.0001)  n.s.  (0.0821) |

## Supplementary Figures


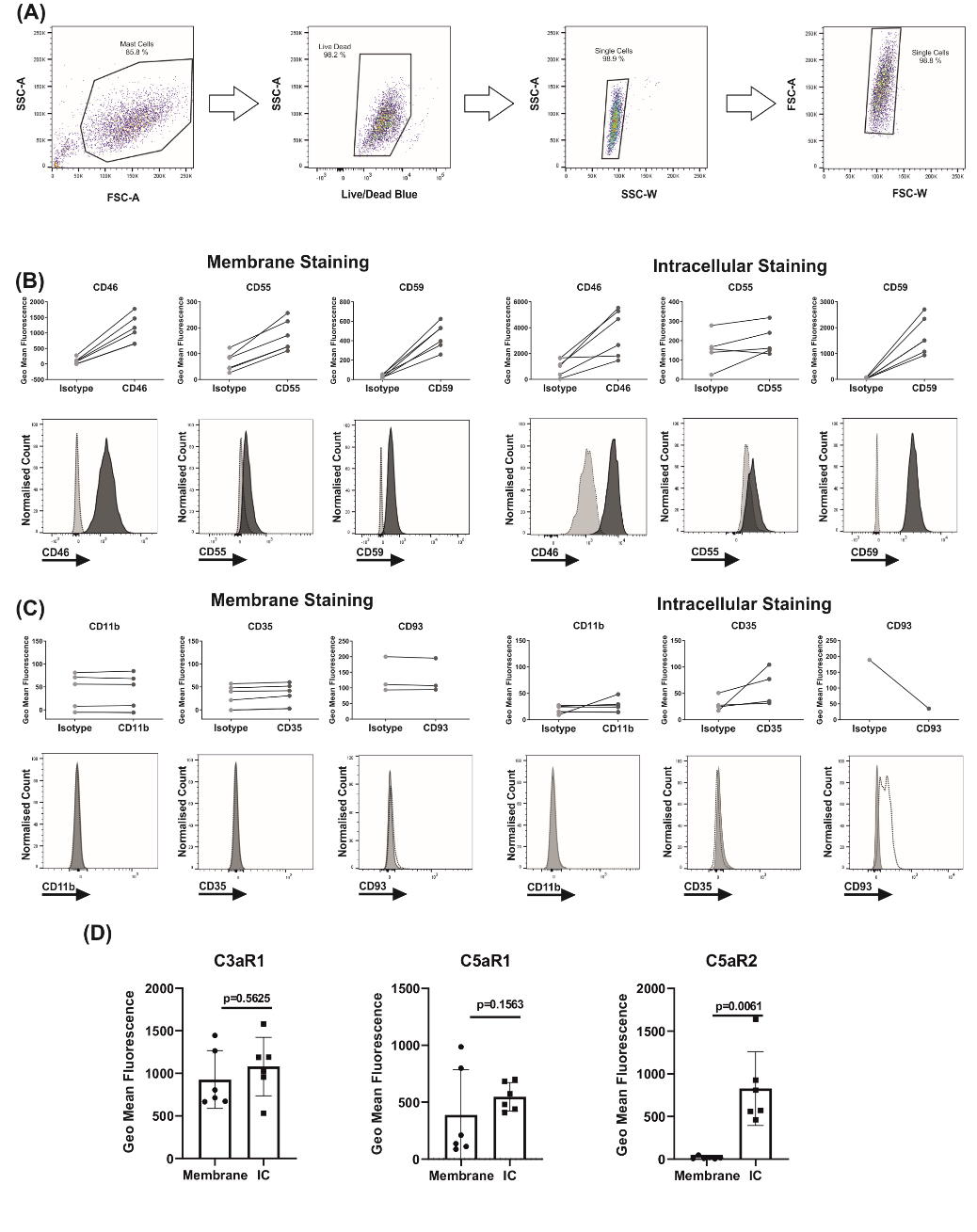


**Supplementary Figure 1. Example gating strategy for peripheral blood derived mast cells and additional complement receptor expression. (A)** A homogenous population of cells consistent with mast cells was selected based on size and granularity (forward- and side-scatter). Dead cells were excluded based on positive inclusion of LiveDead™ blue. Single cells were selected based on area and width parameters in both forward and side scatter. **(B)** Flow cytometric analysis of expression of human complement receptors (dark grey) compared to control (light grey) for CD46, CD55, and CD59 with representative histograms. Data are n=6 from independent experiments on 6 donors. Membrane (left panels) and permeabilised cell (right panels) staining are shown. **(C)** Flow cytometric analysis of expression of human complement receptors (dark grey) compared to control (light grey) for CD11b, CD35 and CD93 on the cell membrane (left panels) or in permeabilised cells (right panels). Data are n=5, 5, 3 and n=5, 4, 1 (CD11b, CD35, CD93 for left and right panels respectively). Representative histograms are shown below with control (dotted line) and antibody stained samples (grey histogram). **(D)** Comparison of geometric mean fluorescence achieved in membrane only stained and permeabilised cell staining (IC). P-values shown obtained from Wilcoxon matched-pairs signed rank test (C3aR1, C5aR1) or paired t-test (C5aR1). Data are and n=6 from independent experiments on separate donors.


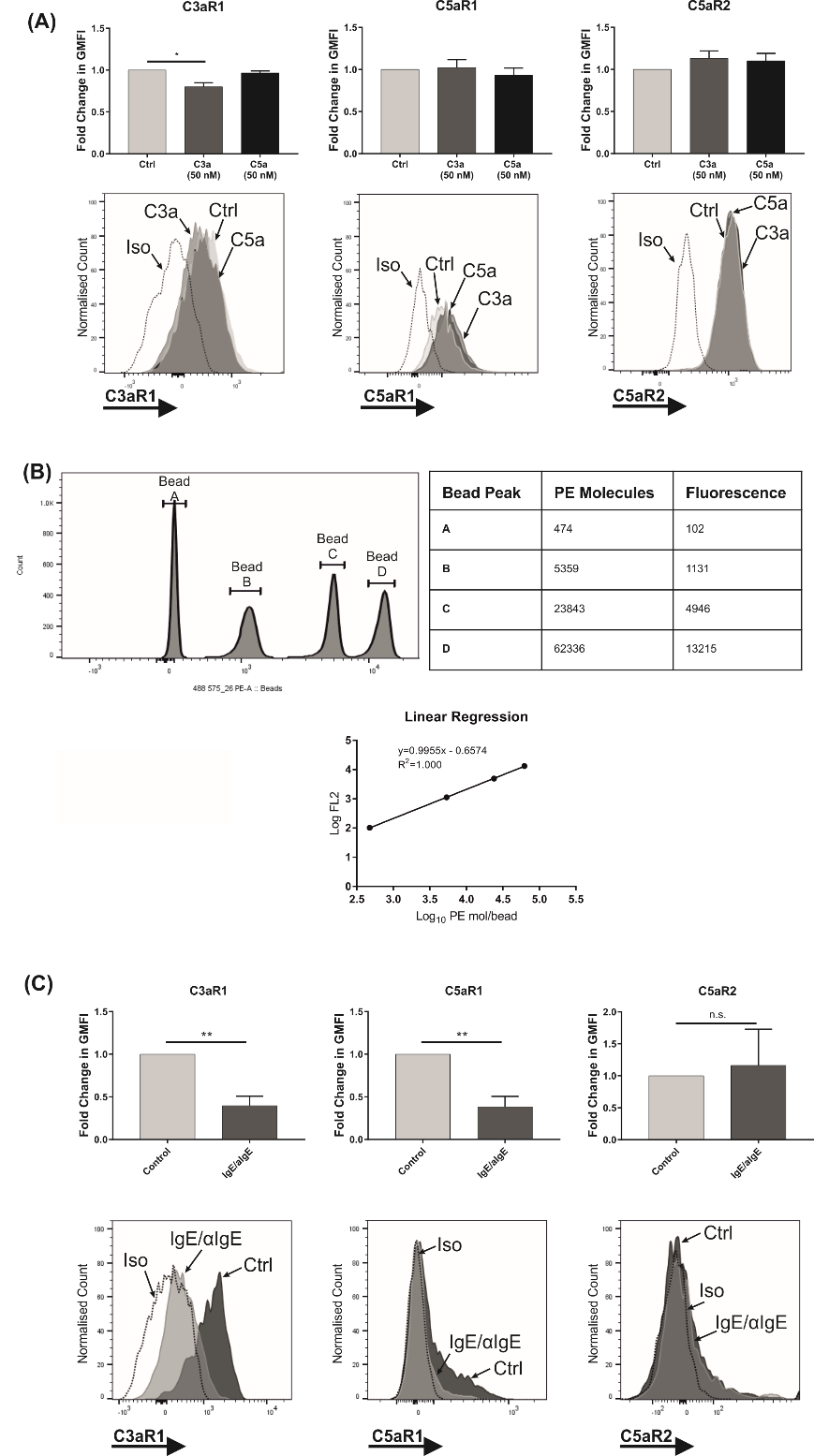


**Supplementary Figure 2. Gating strategy and analysis of receptor expression.** **(A)** Flow cytometric analysis of intracellular expression of complement receptors after addition of ligands C3a (50 nM, dark grey bars), C5a (50 nM, black bars) or media control (light grey bars) for 1 hour. Data are mean ± SEM of n=6 experiments from 6 donors with representative histograms * = p<0.05 (One-way ANOVA with Dunnett's multiple comparison post-test). **(B)** Typical bead peaks used for fluorescence quantitation, demonstrating discrimination of beads, with table of values and log/log linear regression showing the equation for y=mx+c. **(C)** Membrane expression of complement receptors after 1hr stimulation by IgE crosslinking. Data are mean ± SEM of n=5 experiments from separate donors. Significant differences are indicated by *p≤0.05; ** p≤0.01, analysed by paired t-test.


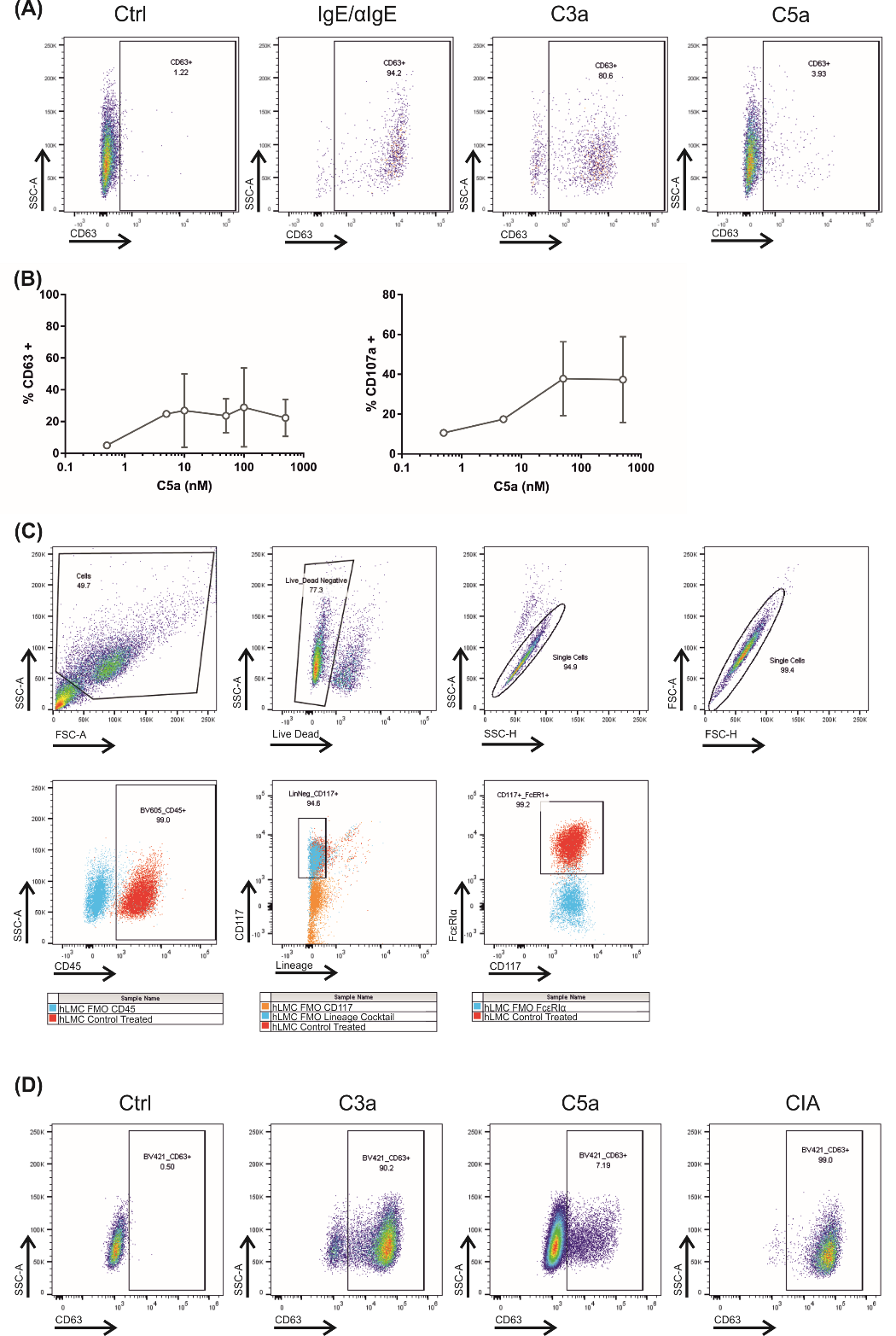


**Supplementary Figure 3.** **Degranulation and hLMC gating. (A)** Example of degranulation of hMC showing CD63 externalisation in response to control, IgE/αIgE (1μg.ml^-1^), C3a (50nM) or C5a (50nM). **(B)** Degranulation in response to varying concentrations of C5a (0-500 nM) shown by externalisation of CD63 and CD107a. Data are mean ± SEM of n=1-3 experiments in separate donors. **(C)** Gating strategy for hLMCs showing CD45+, CD117+/Lineage negative and CD117+/FcεRIα+ cells with FMO controls for each marker. **(D)** Example degranulation of hLMCs showing externalisation of CD63 in response to C3a (50 nM), C5a (50 nM) or CIA23187 (100 nM).


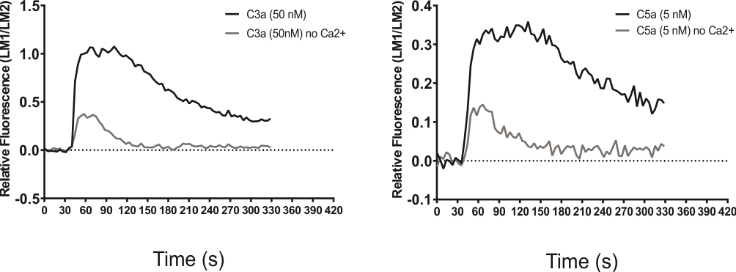


**Supplementary Figure 4. Complement induced calcium flux is dependent on extracellular calcium.** C3a and C5a induced changes in Fura-2 dependent fluorescence over time in the presence of (black line) or absence of (grey line) extracellular Ca2^+^ ions. Data are n=1.

**
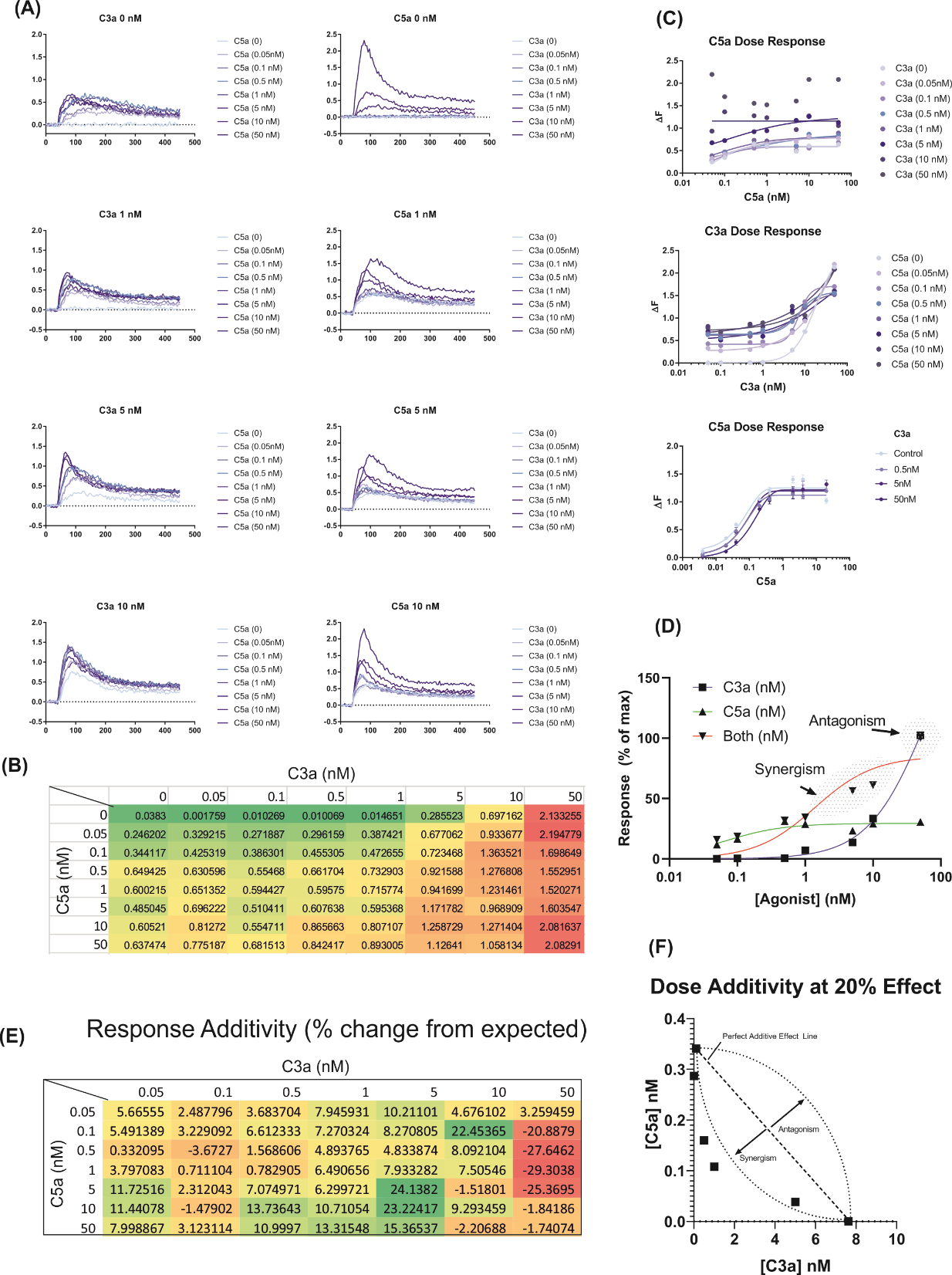
**

**Supplementary Figure 5. Additive effects of complement anaphylatoxins on calcium flux responses.** Calcium specific fluorescence over time **(A)** showing interaction of increasing C5a concentration (0-50 nM) with specific C3a concentrations (0, 1, 5, 10 nM, left hand panels) or interaction of increasing C3a concentration (0-50 nM) with specific C5a concentrations (0, 1, 5, 10 nM, right hand panels). Overall changes in fluorescence (ΔF) raw data at each concentration are shown **(B)**. **(C)** Dose response curves of complement induced change in calcium specific fluorescence showing interaction of C3a and C5a induced calcium flux. **(D)** C3a (0.05-50 nM), C5a (0.05-50 nM) and additive (0.05-50 nM) responses are shown indicating areas of synergism and antagonism in the dose range. **(E)** Response additivity model indicating % synergism or antagonism at each concentration as shown. **(F)** Dose additivity model at 20% maximum effect indicating synergism at low concentration of C5a and C3a. Data are n=1.

**
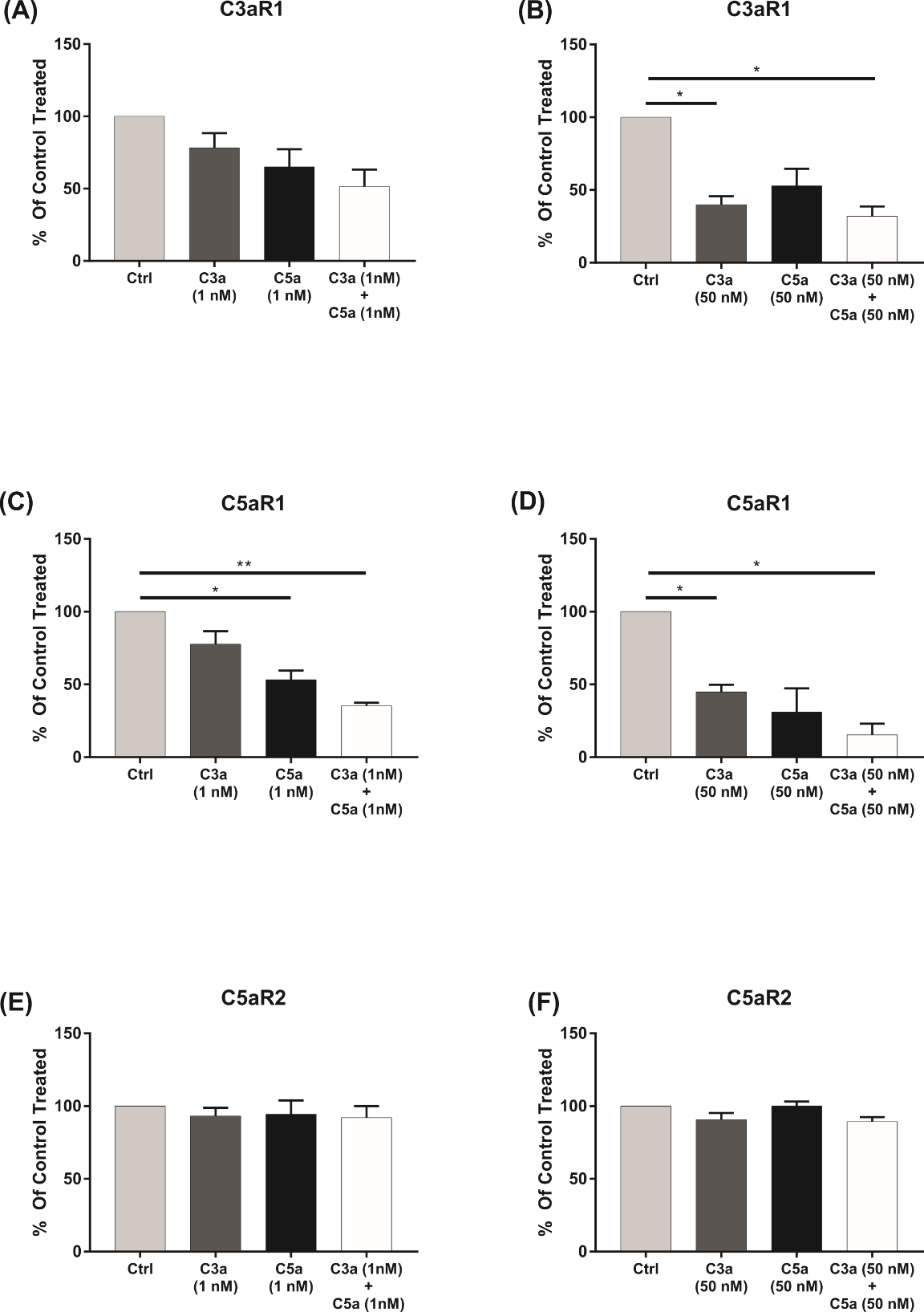
**

**Supplementary Figure 6.** **Modulation of complement receptor expression by combinatorial treatment with complement anaphylatoxins.** Expression of **(A and B)** C3aR1, **(C and D)** C5aR1, **(E and F)** C5aR2 after 8 hours of treatment with 1 nM C3a, C5a or both (A, C, E) or 50 nM C3a, C5a or both (B, D, F). Data are expressed as % change from control treatment and are mean ± SEM of 3 independent experiments of each of 3 pooled donors. Significant differences are indicated by * =p<0.05, **=p<0.01 (one-way repeated measured ANOVA using Tukey's multiple comparison post-test).

**
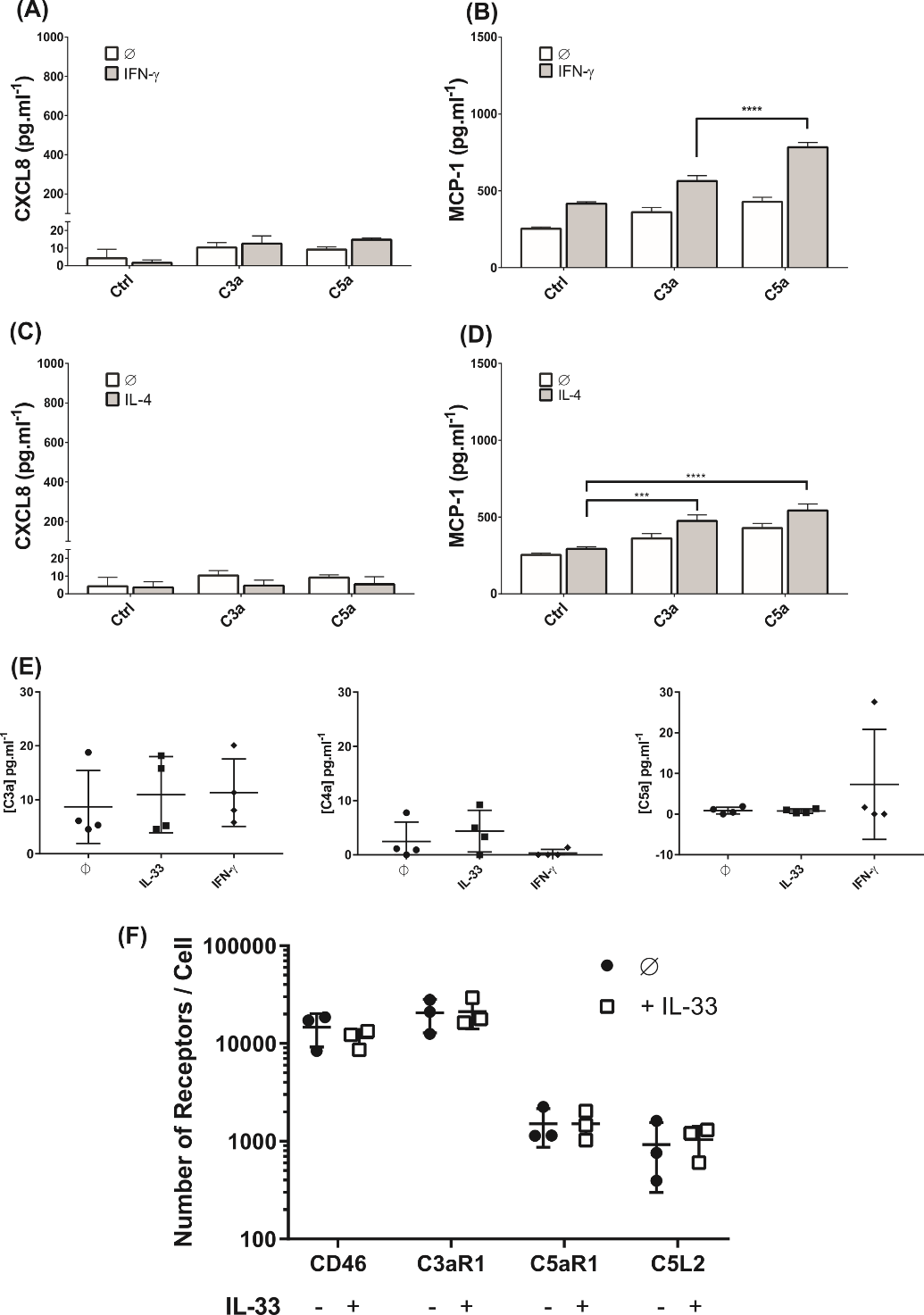
**

**Supplementary Figure 7. Modulation of chemokine and complement anaphylatoxin release from hMC by preconditioning.** Cells pre-treated for 24 hours with media control (white bars), IFNγ (50 ng.ml^-1^, grey bars) **(A)** & **(B)**, or IL-4 (50 ng.ml^-1^) **(C)** & **(D)** followed by activation with control, C3a (50 nM) or C5a (50 nM) for 8 hours. Data are n=3 from 3 replicates of 3 pooled donors. Significant differences are indicated by *** = p<0.00 1, **** = p<0.0001 measured by 2-way ANOVA with Tukey's multiple comparison post-test. **(E)** Complement anaphylatoxin release after preconditioning for 24 hours with media control (circles), IL-33 (50 ng.ml^-1^, squares) or IFNγ (50 ng.ml^-1^, diamonds). Data are n=4 from 4 individual donors. **(F)** Receptor quantification using Quantibrite™ beads after 24 hour incubation with control media (filled circles) or IL-33 (50 ng.ml^-1^) (open boxes). Data are mean ± SD of quantification for 3 separate donors.

**
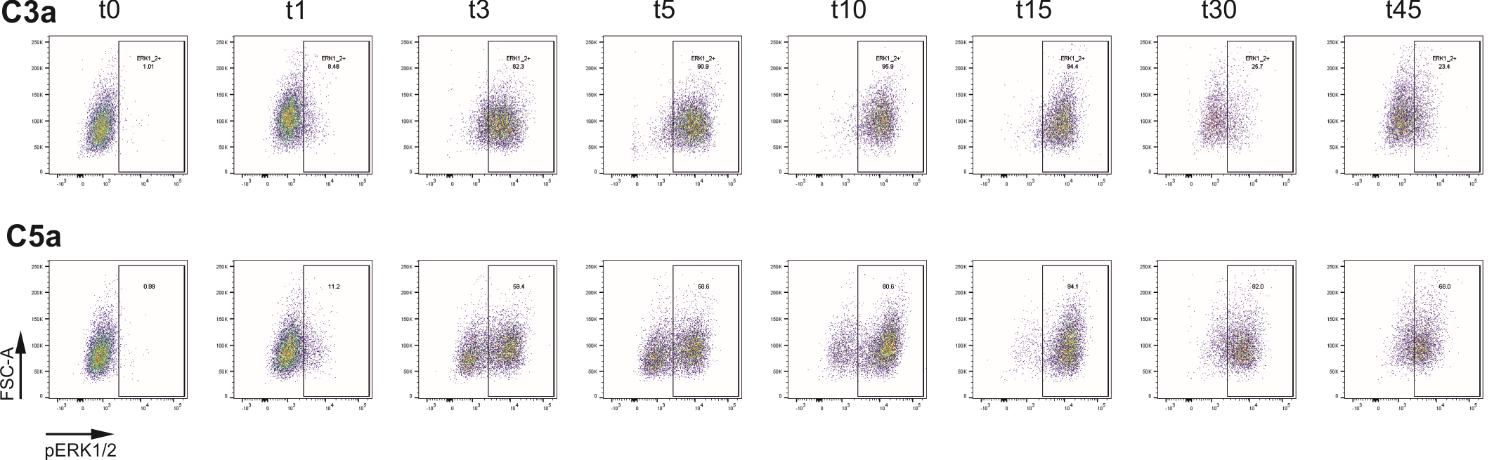
**

**Supplementary Figure 8. Complement anaphylatoxin induced ERK1/2 activation.** C3a and C5a induced ERK1/2 phosphorylation measured by intracellular flow cytometry of samples taken at 0, 1, 3, 5, 10, 15, 30 or 45 minutes post stimulation with 50 nM C3a or C5a. Box shows % of ERK1/2 positive cells at each time point.
